# WHO Environmental Noise Guidelines for the European Region: A Systematic Review on Environmental Noise and Cognition

**DOI:** 10.3390/ijerph15020285

**Published:** 2018-02-07

**Authors:** Charlotte Clark, Katarina Paunovic

**Affiliations:** 1 Acoustics, Ove Arup & Partners, 13 Fitzroy Street, London W1T 4BQ, UK; 2 Institute of Hygiene and Medical Ecology, Faculty of Medicine, University of Belgrade, 11000 Belgrade, Serbia; paunkaya@yahoo.com

**Keywords:** road traffic noise, aircraft noise, railway noise, children, cognition, reading comprehension, memory, attention

## Abstract

This systematic review assesses the quality of the evidence across individual studies on the effect of environmental noise (road traffic, aircraft, and train and railway noise) on cognition. Quantitative non-experimental studies of the association between environmental noise exposure on child and adult cognitive performance published up to June 2015 were reviewed: no limit was placed on the start date for the search. A total of 34 papers were identified, all of which were of child populations. 82% of the papers were of cross-sectional design, with fewer studies of longitudinal or intervention design. A range of cognitive outcomes were examined. The quality of the evidence across the studies for each individual noise source and cognitive outcome was assessed using an adaptation of GRADE methodology. This review found, given the predominance of cross-sectional studies, that the quality of the evidence across studies ranged from being of moderate quality for an effect for some outcomes, e.g., aircraft noise effects on reading comprehension and on long-term memory, to no effect for other outcomes such as attention and executive function and for some noise sources such as road traffic noise and railway noise. The GRADE evaluation of low quality evidence across studies for some cognitive domains and for some noise sources does not necessarily mean that there are no effects: rather, that more robust and a greater number of studies are required.

## 1. Introduction

Recent years have seen an increase in the strength of the evidence linking environmental noise exposure, such as aircraft noise and road traffic noise, to health [1,2,3]. Evidence for effects on children’s wellbeing and learning has also increased in the last decade [4,5,6,7,8,9]. The World Health Organization [10] recently estimated that between 1 and 1.6 million healthy life years (Disability-Adjusted Life Years or DALYs) are lost annually because of environmental noise exposure in high income western European Countries. The World Health Organization estimated that each year 45,000 DALYs are lost due to cognitive impairment in children [10]. The development of cognitive abilities such as reading are important not only in terms of educational achievement but also for subsequent life chances and adult health [11]. Children are often posited to be a group ‘vulnerable’ to the effects of noise [4,12] as this is a time of rapid growth and cognitive development and children might have less developed coping repertoires than adults to deal with environmental noise and less control over noise [4].

Whilst evidence has been increasing, the most influential guidelines for children’s environmental noise exposure, the World Health Organization Guidelines for Community Noise were first published in 1999 [13], prior to much of the available evidence. Focusing on children’s environmental noise exposure, the existing guidelines suggest that school playgrounds outdoors should not exceed 55 dB L_Aeq_ during play to protect from annoyance and that school classrooms should not exceed 35 dB L_Aeq_ during class to protect from speech intelligibility and, disturbance of information extraction. It should however be noted, that 35 dB L_Aeq_ is a very low level of noise exposure, considered unachievable by some stakeholders. These guidelines are currently being revised and updated by the World Health Organization Regional Office for Europe (WHO Europe), specifically for the European Region, who commissioned this systematic review of the evidence to inform their guideline development.

Several plausible pathways and mechanisms for the effects of noise on children’s cognition have been put forward. It has been suggested that noise may directly affect children’s cognitive abilities such as reading comprehension, but effects could also be accounted for by other mechanisms such as teacher and pupil frustration [14]; learned helplessness (low motivation to learn resulting from lack of control over one’s environment) [15]; and increased arousal which can impact task performance. Experimental studies of acute exposure show negative effects on speech perception and listening comprehension [6]. Noise most likely interferes with the interactions between teachers and pupils: teachers may have to stop teaching whilst noise events occur which may contribute to a reduction of morale and motivation in teachers. Another pathway is impaired attention [14,16]. It has also been suggested that children exposed to environmental noise at school may cope with noise exposure by ‘tuning out’ the noise: this strategy may then be over-generalized resulting in poorer learning experiences [17]. Noise exposure causes annoyance, which may lead to physiological and psychological stress responses in some individuals: stress responses are associated with lower mood and performance. Where catchment areas for schools are small, there is a strong correlation between home and school noise exposure [18]: night-time noise might interfere with children’s sleep, which can cause low mood, fatigue and impaired task performance the next day [19].

## 2. Materials and Methods

### 2.1. Scope of the Review

The review sought to identify original research papers of quantitative design, on the effect of environmental noise on cognition published up to June 2015. No restriction on the start date for the search was implemented. Search terms covering different sources of environmental noise (aircraft, road traffic, railway, wind-turbine), different study designs (cross-sectional, longitudinal), and different cognitive outcomes (reading, memory, attention) were included in database searches of Medline/PubMed; Scopus (includes Embase); PsycInfo, Web of Science Database and ScienceDirect. See Web Appendix in Appendix A for the complete list of search terms included. Experimental studies were not included within the scope of the review, as the review focused on the long-term effects of chronic environmental noise exposure on cognition, as opposed to effects of acute environmental noise exposure in the laboratory. For a review of the experimental literature see Klatte et al. (2013) [6].

Several types of cognition were included in the search. Tests of reading and oral comprehension assess various aspects of children’s ability to recognize and use words, literacy, and language. Tests of memory included tests of long-term memory, which is responsible for storage of material for an extended period and has unlimited capacity, and tests of short-term memory, which has a limited capacity and is responsible for the storage of material for a short-time period of time without manipulating it (e.g., verbally repeating a phone number). Tests of working memory and executive function assess being able to keep information in the short-term memory and to manipulate or use it immediately. Standardized assessment tests are used in educational settings to assess a child’s ability in key skills such as English or Maths in relation to their peers or in relation to typical development.

### 2.2. Search Strategy

Quantitative papers in all languages were sought but due to time constraints, conference proceedings were not additionally searched. The reference lists of identified papers were also checked for further relevant citations. Grey-literature, already known to the authors was also included in the review.

### 2.3. Review Process

Papers were reviewed in two stages. First, all the titles and abstracts of the identified papers were reviewed by two reviewers (CC, NS) separately to assess eligibility for inclusion in the review. Second, the full text of eligible papers was retrieved and two reviewers (CC, KP) read the paper and re-assessed eligibility for inclusion. At both stages, where there was disagreement between the reviewers discussion was held until consensus reached.

### 2.4. Data Extraction

Data extraction was undertaken by two reviewers (CC, KP) identifying key features of the study including design (e.g., cross-sectional, longitudinal, intervention), population/setting (e.g., children or adults/home or school), exposure (source, range of exposure, comparison groups), confounding (other factors adjusted for), outcome examined (e.g., which test and which cognitive skill), findings (estimated effect on cognition per 1 dB increase in exposure, where possible) (see supplementary tables). Data extraction tables were compared across the two reviewers and discussed where there was disagreement until consensus was reached.

Each paper was subsequently assessed for the following types of bias:

1. Noise exposure assessment leading to information bias: evaluating whether the paper used established noise metrics in dB; the time-frame of noise measurements, if applicable; quality of noise modelling, if applicable.
2. Bias due to confounding: evaluating whether the study used matching or adjustment in the analysis for potential confounding factors, such as socioeconomic status, which can influence both noise exposure and cognitive performance.
3. Bias due to selection of participants: whether participants are randomly sampled from a known population and whether the response rate was higher than 60%. Consideration of bias associated with drop out for longitudinal studies.
4. Outcome assessment leading to information bias I: whether the cognitive test is objectively measured using a known scale or validated measure.
5. Outcome assessment leading to information bias II: whether the assessment is blinded for exposure information in cohort.

Bias was also considered present for each aspect noted above, if this information was omitted from the paper.

### 2.5. Evaluating the Quality of the Evidence

The study design and methods used within each cognitive ability domain varied widely in terms of noise exposure (and how defined) and in terms of which test of cognition had been employed and how effects had been estimated. For these reasons, this is a narrative systematic review, rather than a systematic review including meta-analyses. Unfortunately, the studies are not uniform in how they define exposure or in how they measure specific cognitive abilities to enable meta-analyses within each cognitive ability domain.

In order to assess the quality of the evidence for each health outcome required for appropriate recommendations, we used the GRADE methodology which ranks the quality of evidence as high, moderate, low, or very low [20]. GRADE is the methodology recommended by the WHO Handbook for Guideline Development [21]. This review adapted the GRADE methodology, which traditionally assigns high quality evidence only to evidence from studies of a randomized control study design: this is inappropriate for this field where the authors felt that studies of a longitudinal design should be assigned the highest quality of evidence available. Where only cross-sectional studies were available the evidence was initially judged as being of low quality.

The GRADE methodology allows for these initial evidence ratings to be further upgraded or downgraded according to specific criteria [21]. Upgrades can be made based on the availability of evidence for an exposure-response relationship between noise and cognition; the magnitude of the relative risk being >2; or there being evidence for an effect in spite of confounding working towards the null. Downgrades can be made based on most of the studies being of low quality (study design); inconsistent findings between studies (inconsistency); studies not comparing the same outcomes (indirectness); effect estimate confidence interval containing 25% harm or benefit (precision); or publication bias, as assessed by a funnel plot. Unfortunately it is not possible to assess precision and publication bias when undertaking a narrative review.

## 3. Results

### 3.1. Papers Identified

**Figure 1:**
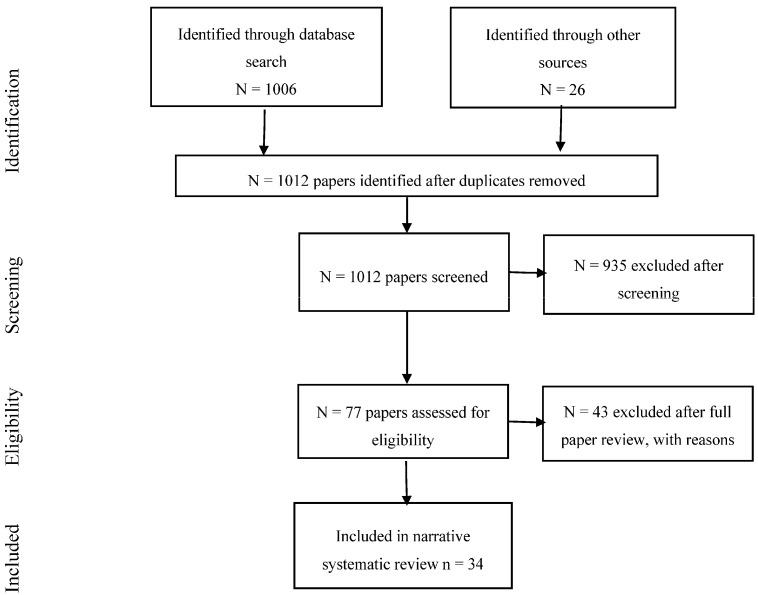
Flow chart showing the review process for cognition.

In total, 1006 citations were identified from a search of the databases. Following this systematic process of searching for papers, the authors felt that some key older papers from the field had not been retrieved by the database searches and added a further 26 papers to the data extraction process. These papers were identified from reference lists of existing narrative reviews and were papers identified by the authors undertaking the review, relating to older or very recent studies and reports in the field. The authors have published previous narrative reviews of the field [1,2,3,4,22] so were familiar with the existing literature. It is thought that the database searches missed some of these papers as they were older or because they were reports not published in peer-reviewed journals. After removing duplicates, 1012 papers were identified from the database search and the additional papers. Screening of the citations identified 77 that were potentially relevant, of which 34 were included and 43 were excluded after full text retrieval. Reasons for exclusions included that the study did not measure noise exposure or cognition, that it was a review paper, or an experimental study. This led to a total of 34 primary research papers for inclusion in the review (see Figure 1).

### 3.2. Summary of Papers

Table 1 summarizes the key features of the papers identified in terms of study design, noise exposure characterization, and cognitive outcomes. The majority of the studies were of cross-sectional design (82%); there were far fewer studies of more robust quantitative design such as longitudinal studies (21%) and intervention studies (15%). All of the studies were of children, with most studies sampling in mid-childhood (aged 8–12 years).

Most studies examined aircraft noise exposure (74%), with a further 11 studies examining road traffic noise exposure (32%). Few studies examined rail noise or ambient or background noise exposure, and there were few studies of noise and co-occurring air pollution exposure. The majority of studies focused on noise exposure in the school environment (88%), as opposed to the home environment (35%), using annual average noise exposure metrics (L_Aeq_− 79% or L_dn_ 12%). Few studies examined other metrics such as L_Amax_.

A range of cognitive abilities had been examined. The most commonly reported were tests of reading and oral comprehension as assessed by direct testing (41%) or Standardised Achievement Test (SATs) data (38%). Studies had also assessed short-term and long-term memory (35%); attention (38%); and working memory/executive function (26%).

Most of the studies took adequate account of sociodemographic confounding between noise exposure and cognitive performance, but it should be borne in mind that older studies from the 1970s and 1980s are considerably less likely to have taken socioeconomic confounding into account. Also, the studies included in this review, are not always independent: for example, there are several publications of the RANCH data, making use of the same data, but examining noise exposure at different times of the day or making further adjustments for air pollution or examining sub-samples.

### 3.3. Evaluating the Quality of the Evidence

The following sections summarize the quality of the evidence for environmental noise effects on reading and oral comprehension, impairment assessed through standardized assessments such as SATs, short-term and long-term memory, attention, and executive function deficit (working memory, capacity etc.). The GRADE methodology is used to assess the quality of the evidence across the evidence base for each environmental noise source and outcome. Initially, each individual study is assessed for several types of bias associated with the study design; noise exposure assessment; confounding/adjustment; selection of participants; and the outcome assessment. GRADE then reviews the number of studies in the field; the findings for different noise sources; the consistency of findings across studies; the study design (e.g., whether there is longitudinal evidence or intervention evidence versus cross-sectional evidence); whether there is evidence for exposure-response relationships between noise exposure and the cognitive outcome; and the presence of bias in the available evidence. The GRADE methodology is used to assess the quality of the evidence across the available evidence base for each environmental noise source and cognitive outcome. It is important to note that all studies are individually assessed for the risk of bias and then evaluated using the GRADE system for the quality of evidence; the GRADE assessment is, thus, based on the assessment across the studies/evidence base available for the specific noise source and outcome [21]. An overview of the ratings for the quality of the evidence for the different cognitive domains is given in Table 2.

### 3.4. Tests of Reading and Oral Comprehension

We identified 14 studies of noise effects on tests of reading and oral comprehension [18,23,24,25,26,27,28,29,30,31,32,33,34,35]. Of the studies identified, 11 were cross-sectional, with only four longitudinal [27,29,30,32] and Two interventions studies identified [30,32] both of which examined the relocation and/or opening of a new airport. Most studies comprised European, North American and African children, usually aged 8–12 years. The detailed data extraction for each of these studies is given in Supplementary Table S1 organised by study design.

Due to differences in language across countries, the studies often use different tests of reading comprehension, albeit, usually nationally standardised tests within each country. Several English speaking countries have employed the same test of reading comprehension—the Suffolk Reading Scale, which has been used by the UK sample of the RANCH study [18], as well as in South Africa [32,33], and in other UK studies [23,24,31].

The majority of studies were of aircraft noise exposure (n = 14), with only two papers examining the effects of road traffic noise (this is the same study reported in two papers) and no studies examining the effects of railway noise.

The GRADE evaluation of these papers is given in Table 3. The risk of bias was judged to be low in these individual studies. Whilst the evidence was predominantly from studies of cross-sectional design, with few intervention or longitudinal studies, noise exposure assessment was based upon long-term measurement data using established metrics, airport contour data, or good quality noise modeling. Studies made good adjustment for socioeconomic and other confounders and participants were are usually identified by the random selection of schools within a geographical area, with good response rates. Reading or oral comprehension was assessed using standardized and established tests.

#### 3.4.1. Aircraft Noise Exposure

There were 14 studies that examined aircraft noise exposure effects on reading and oral comprehension [18,23,24,25,26,27,28,29,30,31,32,33,34,35]; 11 were cross-sectional, four were longitudinal, and two were intervention studies (relocation or opening of a new airport). Of these 14 studies, 10 demonstrate a statistically significant association between higher aircraft noise exposure and poorer reading comprehension [18,24,25,26,28,29,30,33,34,35]. Two further studies suggest a trend for aircraft noise exposure to influence reading comprehension [23,27]. Two studies found no statistically significant association between aircraft noise exposure and reading comprehension [31,32]. Only one study has demonstrated exposure-response relationships for aircraft noise exposure [18].

Applying the GRADE framework to assess the quality of evidence across the available studies of aircraft noise on reading and oral comprehension (Table 3), we considered longitudinal or intervention studies the ideal study design. We downgraded the evidence to moderate evidence based on some inconsistency in findings across studies. We therefore concluded that there was moderate quality evidence regarding an effect of aircraft noise on reading and oral comprehension.

#### 3.4.2. Road Traffic Noise Exposure

Two publications of the same study [18,26] of road traffic noise and tests of reading and oral comprehension were identified. No longitudinal or intervention studies were identified. There is no evidence from these cross-sectional studies that road noise exposure is associated with children having poorer performance on tests of reading and oral comprehension.

For the quality of evidence across the studies of road traffic noise and reading and oral comprehension, adapting the GRADE approach, we considered longitudinal or intervention studies the ideal study design. We therefore initially judged the evidence to be of low quality based on the lack of longitudinal and intervention studies, as the only studies available were cross-sectional (Table 4). This was further downgraded to very low quality as we were unable to assess consistency across different studies. We found no reason to further upgrade the evidence and therefore concluded that there was very low quality evidence for no substantial effect of road traffic noise on reading and oral comprehension.

#### 3.4.3. Railway Noise Exposure

We were unable to assess the evidence using the GRADE approach, as no studies of railway noise exposure on tests of reading and oral comprehension were identified.

### 3.5. Impairment Assessed through Standardized Assessments (SATs)

We identified 13 studies of noise effects on impairment assessed through standardized assessments such as SATs [16,36,37,38,39,40,41,42,43,44,45,46]. The studies were of European and North American samples, and covered a range of ages from early- and mid-childhood to adolescence. See Supplementary Table S2 for the detailed data extraction for each of these papers. The studies examined a range of noise exposures: seven were of aircraft noise exposure; four were of road traffic noise; and two were of railway noise. The results of the GRADE evaluation in terms of the overall quality of evidence is given in Table 4.

The risk of bias in these individual studies was judged to be high. Whilst noise exposure assessment, selection of participants and outcome assessment were judged to be of good quality, bias due to confounding is likely due to the study design. Whilst, the studies either match samples or make adjustment for socioeconomic and other confounders, these confounders are from data at the school-level, not the individual-level, meaning that residual confounding by socioeconomic factors may remain. Further, some of the older studies, from the 1970s, fail to make any or adequate adjustment for confounding.

#### 3.5.1. Aircraft Noise Exposure

We identified seven studies of aircraft noise exposure and national standardized test scores (SATs), of which three were intervention studies [39,40,43] of sound insulation or airport relocation, two were longitudinal [17,38] and two were cross-sectional [42,46]. Of these seven studies, four studies suggest a significant association between higher aircraft noise at school and poorer SATs scores [39,40,42,43], and three studies suggest no significant association between higher aircraft noise exposure at school and SATs scores [17,38,46]. Two studies, of the same data, provide equivocal evidence, with evidence for a negative association, for no association, and for a positive association between higher aircraft noise exposure and SATs scores all demonstrated within the same study [39,40]: these findings may be explained by the small sample and low power in this study. The two intervention studies both suggest that sound insulation is associated with an improvement in SATs scores [39,40,43].

For the quality of evidence across the available studies of aircraft noise effects on standardized assessment tests, adapting the GRADE approach, we considered longitudinal or intervention studies the ideal study design and designated such evidence as high quality (Table 4). However, this was downgraded to moderate quality, due to the rating of high risk of bias associated with residual socioeconomic confounding. We therefore concluded that there was moderate quality evidence for an effect of aircraft noise on standardized assessment tests.

#### 3.5.2. Road Traffic Noise Exposure

We identified four cross-sectional studies of road traffic noise exposure and national standardized test scores (SATs) [16,41,44,45]. No studies were intervention or longitudinal studies. All four studies suggest associations between road traffic noise exposure at school or at home and performance on SATs. One study, included assessments of both external and internal road traffic noise exposure in the classroom, finding associations of external and internal road traffic noise in relation to national test scores for primary school children aged 7–11 years, even after adjustment for socioeconomic status [41]. The study also found the strongest associations for test scores was with L_Amax_ metrics for road traffic noise, which suggests that individual noise events may be important.

For the quality of evidence across the available studies of road traffic noise effects on standardized assessment tests, as we only had evidence from cross-sectional studies, we started with a rating of low quality evidence (Table 4). This was downgraded to very low quality based on potential bias associated with residual confounding by socioeconomic status at the individual level. We therefore concluded that there was very low quality evidence for an effect of road traffic noise on standardized assessment tests.

#### 3.5.3. Railway Noise Exposure

We identified two studies which examined the association between railway noise exposure and SATs scores [36,37]: one of these was an intervention study of sound insulation [36]: and one was a cross-sectional study [37]. Both studies suggest an association of railway noise exposure at school on SATs scores, with the intervention study suggesting that the effect disappeared once noise abatement work had been undertaken.

For the quality of evidence across the available studies of railway noise effects on standardized assessment tests, we evidence from one intervention study, we would designate the start level for this evidence as high quality (Table 4). As there was a high risk of bias across the individual studies associated with residual socioeconomic confounding, we downgraded this rating to moderate quality evidence. We therefore concluded that there was moderate quality evidence for an effect of railway noise on standardized assessment tests.

### 3.6. Short-Term and Long-Term (Episodic) Memory

We identified 12 studies of noise effects on tests of short-term and long-term (episodic) memory [23,24,25,26,28,30,31,35,47,48,49,50]. The studies were of European and North American samples. 11 studies were cross-sectional. Only one study was longitudinal and of an intervention of an airport closure/relocation [30]. Most studies examine long-term (episodic) memory, with a range of different outcome measures being used across the studies. These tests not only often differ between studies but can also differ in terms of what specific aspect of memory was being assessed. Many studies focus on children’s recall of material presented via stories: such tests of memory assess either intentional memory—where the child is informed that they will be tested on the content of the story (e.g., recognition memory, information recall), or incidental memory—where the child is unaware that they will later be subsequently tested on the material [48]. The studies examined a range of noise exposures including aircraft noise exposure, road traffic noise and railway noise. The data extraction for these studies is in Supplementary Table S3. The GRADE evaluation of these papers is given in Table 5.

The risk of bias was judged to be low for these individual studies. Whilst the evidence was predominantly cross-sectional, with few intervention or longitudinal studies, noise exposure was based on long-term measurement data using established metrics, airport contour data, or good quality noise modeling, the studies made good adjustment for socioeconomic confounding, and were selected randomly from schools or homes within a geographical area, with good response rates. Established, age-appropriate tests of memory had been employed.

#### 3.6.1. Aircraft Noise Exposure

We identified 11 studies that examined the association of aircraft noise exposure on children’s long-term or short-term memory [23,24,25,26,28,30,31,35,47,48,49,50]. Only one study was of an intervention and was longitudinal [30] assessing airport closure/relocation. Of these 11 studies, six found an association of aircraft noise exposure on children’s memory [23,25,26,28,30,49], whilst five studies found no statistically significant association [24,31,35,47,50]. Of the five studies finding an association, only one was an intervention and longitudinal study [30]. Several of these studies analyzed the RANCH project data [25,26,28,49], finding significant associations for some types of long-term memory tasks but not demonstrating statistically significant associations uniformly across different memory tasks.

For the quality of the evidence available across the studies of aircraft noise effects on long-term and short-term memory, having identified one intervention study, we started by designating the evidence as high quality (Table 5). The evidence was downgraded to moderate quality considering the inconsistent findings across the studies. We found no reason to upgrade the evidence. We therefore concluded that there was moderate quality evidence for an effect of aircraft noise on long-term and short-term memory.

#### 3.6.2. Road Traffic Noise Exposure

We identified five cross-sectional studies reported on associations between road traffic noise exposure and children’s long-term memory [26,28,47,49,50]. These studies all report on the RANCH study data. Three of these five studies suggest no association between road traffic noise exposure and children’s long-term memory [28,47,50]: the other two suggest no associations for some long-term memory outcomes, but positive associations for some long-term memory outcomes [26,49].

For the quality of the evidence available across the studies of road traffic noise effects on long-term and short-term memory, as only cross-sectional evidence was identified, the evidence was designated low quality (Table 5). The evidence was downgraded further to very low quality considering the inconsistent findings. We found no reasons to upgrade the evidence. We therefore concluded that there was very low quality evidence for an effect of road traffic noise on long-term and short-term memory.

#### 3.6.3. Railway Noise Exposure

One cross-sectional study reported on railway noise exposure associations with children’s long-term memory [48]: this study suggested a significant association of railway noise with long-term memory performance.

For the quality of the evidence available across the studies of railway noise effects on long-term and short-term memory, one cross-sectional study was identified so the evidence was graded as low quality (Table 5). This rating was downgraded to very low quality evidence since we cannot assess consistency of findings, as there is only one study. No further reasons to downgrade or upgrade the evidence were identified. However, this conclusion should be tempered by the fact that it is drawn from the findings of only one study. We therefore concluded that there was very low quality evidence for an effect of railway noise on long-term and short-term memory.

### 3.7. Attention

We identified 13 studies which examined the association of environmental noise exposure on tests of attention [16,17,24,25,26,29,30,31,38,47,48,50]. One of these studies was an intervention study of airport closure/relocation [30] but the majority were cross-sectional studies. The studies were predominantly of children in mid-childhood (8–11 years). The studies use a range of different tests of attention. The majority of studies assessed aircraft noise (n = 10), with only five studies examining road traffic noise and one study examining railway noise. Supplementary Table S4 gives the detailed data extraction for these studies. The results of the GRADE evaluation in terms of the overall quality of evidence is given in Table 6.

The risk of bias was judged to be high for some of these individual studies for aircraft and road traffic noise: these were older studies that did not adjust for confounding and potentially had information bias associated with noise assessment. The risk of bias was rated as low for railway noise, where only one more recent study was available. Whilst the evidence was predominantly cross-sectional, noise exposure was based on long-term measurement data using established metrics, airport contour data, or good quality noise modeling, the studies made good adjustment for socioeconomic confounding, and were selected randomly from schools or homes within a geographical area, with good response rates. Established, age-appropriate tests of attention had been employed. However, bias was judged to be high for aircraft and road traffic noise studies given potential information assessment leading to bias. Further, many of the studies, which compare a ‘high’ exposure with a ‘low’ exposure group, use different thresholds, so ‘high’ noise exposure in the studies covers a range of different exposures.

#### 3.7.1. Aircraft Noise Exposure

We identified 10 studies which examined associations of aircraft noise exposure with tests of attention [17,24,25,26,29,30,31,38,47,50]. There was one intervention study of an airport closure/relocation [30] and two longitudinal studies [29,30]: leaving eight cross-sectional studies. Of these 10 studies, five, including one longitudinal study, found a statistically significant association between aircraft noise exposure and tests of attention [17,29,38,47,50], whilst five studies, including the only intervention study, found no statistically significant association between aircraft noise and attention [24,25,26,30,31].

For the quality of the evidence available across the studies of aircraft noise effects on attention, given the evidence from longitudinal or intervention studies, the evidence was initially designated as high quality (Table 6). This was downgraded to low quality evidence given the inconsistent findings across the studies. We therefore concluded that there was low quality evidence for no substantial effect of aircraft noise on attention. 

#### 3.7.2. Road Traffic Noise Exposure

We identified five studies that examined the association of road traffic noise exposure with tests of attention [16,26,47,50,51]. All of these studies were cross-sectional. Of these five studies, two studies found a statistically significant association between road traffic noise exposure and attention, albeit on the same data [47,50]; one study found mixed evidence for an association (one assessment showed an association, whilst another assessment did not) [51]; two studies found no statistical association between road traffic noise and attention [16,26].

For the quality of the evidence available across the studies of road traffic noise effects on attention, as only cross-sectional evidence was available, the evidence was initially designated as low quality (Table 6). This was further downgraded to very low quality evidence given the inconsistent findings across the studies. We therefore concluded that there was very low quality evidence for no substantial effect of road traffic noise on attention.

#### 3.7.3. Railway Noise Exposure

We identified one cross-sectional study that reported on railway noise exposure associations with children’s attention [48]. Examining modest levels of ambient community noise (mainly train and rail noise) this study found no difference in 9–10 years old children’s performance on a visual search test of attention for children exposed to noise levels above 60 dBA, compared with children exposed to ambient noise levels below 50 dBA [48].

For the quality of the evidence available across the studies of railway noise effects on attention, as only one cross-sectional study was identified, the evidence was initially designated as low quality (Table 6). This was further downgraded to very low quality evidence as consistency in results cannot be assessed. No reasons to upgrade the evidence were identified. However, this conclusion should be tempered by the fact that it is drawn from the findings of only one study. We therefore concluded that there was very low quality evidence for no substantial effect of railway traffic noise on attention.

### 3.8. Executive Function Deficit (Working Memory Capacity)

We identified nine studies that examined the association of environmental noise exposure on tests of executive function deficit [23,24,25,26,28,35,47,49,50]. These studies mainly use tests of working memory. All the studies were cross-sectional. The studies used a range of different tests of executive function—mainly assessing working memory function.

All nine studies examined aircraft noise exposure, with five of these studies also examining road traffic noise (albeit, all using the same data) [26,28,47,49,50]. No studies were identified of railway noise exposure. Supplementary Table S5 gives the detailed data extraction for these studies. The GRADE evaluation of these studies is in Table 7.

Despite there being no intervention or longitudinal studies, noise characterization was based on long-term measurement or modeling, with sample selection and adjustment for socioeconomic confounding. Age appropriate, established tests of executive function were used. However, noise characterization was more problematic for the aircraft studies, as the studies used different thresholds to define ‘high’ aircraft noise exposure.

#### 3.8.1. Aircraft Noise Exposure

Of the nine cross-sectional studies examining the association of aircraft noise exposure on tests of executive function deficit none report a significant association [23,24,25,26,28,35,47,49,50].

For the quality of the evidence available across the studies of aircraft noise effects on executive funding, we considered longitudinal or intervention studies the ideal study design and would designate such evidence as high quality. However, only cross-sectional studies were available, so the start point for the GRADE evaluation was low quality evidence (Table 7).

This was downgraded to very low quality evidence given the bias associated inconsistency from the use of different thresholds to define ‘high’ noise exposure in the different studies. We found no reasons to upgrade the evidence. We therefore concluded that there was very low quality evidence for no substantial effect of aircraft noise on executive function.

#### 3.8.2. Road Traffic Noise Exposure

We identified five cross-sectional studies that examined associations of road traffic noise exposure on executive function: however, these studies all report on the same data from the RANCH study [26,28,47,49,50]. None of the studies report a significant association.

For the quality of the evidence available across the studies of road traffic noise effects on executive functioning, adapting the GRADE approach, we considered longitudinal or intervention studies the ideal study design and would designate such evidence as high quality. However, only cross-sectional studies were available, so the start point for the GRADE evaluation was low quality evidence (Table 7). No reasons to further downgrade or upgrade the evidence were identified. We therefore concluded that there was low quality evidence for no substantial effect of road traffic noise on executive function.

#### 3.8.3. Railway Noise Exposure

We were unable to assess the evidence using the GRADE approach, as no studies of railway noise exposure on tests of executive function were identified.

## 4. Discussion

This systematic review has assessed the quality of the evidence across the available studies for aircraft noise exposure, road traffic noise exposure, and railway noise exposure on a range of children’s cognitive abilities. The review has also enabled the identification of gaps in knowledge and important areas for further research. Overall, in assessing the quality of the evidence across the available studies we drew the following conclusions from this review based on using the GRADE methodology.

There was moderate quality evidence across the available studies for an effect of aircraft noise on children’s reading and oral comprehension. There was very low quality evidence for no substantial effect of road traffic noise on children’s reading and oral comprehension. Studies of other noise sources, such as railway noise, on children’s reading and oral comprehension are lacking. Further studies of road traffic noise exposure would also prove useful.

There was moderate quality evidence across the available studies regarding an association of aircraft noise and railway noise, and very low quality evidence regarding an association of road traffic noise exposure with poorer performance on standardized assessment tests.

There was moderate quality evidence across the available studies regarding aircraft noise being associated with children having poorer long-term memory. Evidence for an effect of road traffic noise and for railway noise was rated as very low quality. There was a lack of studies examining effects on short-term memory.

There was low quality evidence across the available studies for no substantial effect of aircraft noise on children’s attention. Evidence for no substantial effect of road traffic noise and railway noise was rated as very low quality. However, across the studies there is equivocal evidence for and against an effect of aircraft noise, road traffic and railway noise being associated with children’s attention.

There was very low quality evidence across the available studies for aircraft noise and low quality evidence for road traffic noise for no substantial effect on executive function (working memory), with studies consistently suggesting no association for aircraft noise or road traffic noise. No studies of railway noise were identified.

It is important to appreciate that the GRADE methodology assesses the quality of the evidence available across the evidence base identified: a GRADE rating of no effect or low quality evidence does not directly apply to individual studies. An individual study may show an association of a specific noise exposure with a specific outcome, but evaluating the association across a number of studies, a different conclusion may be reached using the GRADE methodology. There may and are differences between evidence available at an individual study level and evidence available across the field for a specific noise source and cognitive outcome. It is also important to appreciate that a GRADE rating of ‘low quality evidence’ is given by default to all studies with a cross-sectional study design. It in no way implies that the authors of this systematic review consider the study to be of ‘low quality’.

Key limitations of the available evidence include a lack of evidence from longitudinal and intervention studies across all of the cognitive outcomes. The field of research would benefit from research investment in these study designs, to further knowledge about the long-term consequences of environmental noise exposure for children and to inform the design of effective interventions to reduce noise exposure for children. There is also a lack of studies examining exposure-response relationships across the different cognitive domains. Most studies tend to examine children in middle childhood (aged 8–12 years) and future studies would benefit from examining the effect of environmental noise exposure on the cognition of younger children including infants, and adolescents. Further, we identified no epidemiological studies of adult populations and this remains a research priority for future research.

The papers identified almost all focus on using noise metrics based on average sound pressure levels over a given period of time, such as the day-time or night-time period. There is debate within the field as to about whether aggregated noise measures such as these L_Aeq_ measures best represent how human’s respond to noise and about how best to conceptualise the ‘dose’ of exposure in community studies of noise exposure. Other noise metrics need to be explored in relation to cognitive outcomes but some recent studies have found effects on cognition using metrics such as L_Amax_ (the highest sound pressure level in a given time-period) as well as with L_Aeq_ measures [41,43].

A major limitation to this systematic review is the lack of studies in many of the domains studied. It is difficult to draw conclusions specifically about the quality of the evidence across the evidence base when there are few studies. We were unable to assess the strength of the association or the effect size due to the narrative way the study results were combined. The GRADE conclusions regarding very low quality or low quality evidence for noise effects across studies in some cognitive domains does not necessarily mean that there are no effects: rather, that more studies are required. The conclusions of this systematic review broadly agree with recent narrative reviews of environmental noise effects on children’s cognition [1,2,4] which have concluded that there is evidence for an effect of noise exposure at school on children’s cognitive skills such as reading and memory, as well as on standardised academic test scores. However, this current systematic review, which differs in considering the evidence for each noise source (aircraft, road, railway) separately, identifies that the quality of the evidence is currently stronger for some noise sources and outcomes than for others. In particular, previous reviews have relied on evidence from studies of aircraft noise exposure to support the conclusion of effects of environmental noise exposure, per se. This is not unreasonable, given that there are few studies available of railway and road traffic noise exposure for some cognitive outcomes. This approach is often taken in health impact assessments when evidence for a specific noise source is lacking.

The review does not take into account evidence published after July 2015. As with any review, additional publications for some sources and outcomes have the potential to alter some of the conclusions of the current review, particularly for fields where there are currently relatively few papers. Further, given that the authors’ added 26 papers to the review after a database search, it remains possible that further papers may have been omitted from this systematic review [52]. Several studies of adults have been published whilst this review has been ongoing which would have made an important addition to the review [53,54,55].

It is important to note that the different cognitive domains underpinning this review were provided at the outset to the authors by the Guideline Development Group (GDG) undertaking the revision of the WHO Community Guidelines. The domains were not reorganized or amended or added to as part of the review process. The review is not able to address the complex relationship that exists between the different cognitive domains under consideration. For example, we may have been justified in combining the studies identified within the domain of reading comprehension and oral comprehension, with those identified in the domain of SATs that also assessed reading or literacy skills. Further, the review makes a false dichotomy between different cognitive skills that are likely to be related such as attention and short-term memory, and reading and executive function. Cognitive skills do not exist, nor are they employed, in isolation. See the following papers for further useful discussion of these issues [56,57,58].

Community studies of environmental noise effects on cognition conceive of the effects on cognition to be an after-effect of exposure to environmental noise over a certain ‘chronic’ period of time. They do not focus on immediate effects of environmental noise exposure on cognition: i.e., how noise might immediately influence cognitive performance. Immediate effects of noise might be particularly important for attentional outcomes and the scope of this review has meant that these immediate effects have not been taken this into account. We have not assessed the strength of the evidence for immediate effects of noise on cognition, which is examined using an experimental approach.

Another major limitation to the review is the lack of homogeneity of methods and reporting between the studies, even within cognitive domains, which has meant that it has not been possible to conduct meta-analyses across the studies. Such meta-analyses would enable the effect across studies to be estimated, which would inform uncertainty relating to the study findings. Unfortunately, this is not yet possible for several reasons. Studies often use different tests of the same cognitive ability: combining estimates across studies that use different outcomes is challenging and often not possible. Further, many studies group exposure into high and low, using different thresholds for high and low, which again makes combining study data challenging as the range of noise exposure within the high and low categories is often unknown and cannot be estimated reliably from the data provided. The potential to be able to conduct meta-analyses within this field will be greatly enhanced if future studies report effect estimates for a 1 dB and 5 dB increment in noise exposure and if studies report the range of noise exposure in their population even if their design involves selecting samples based on high and low noise exposure. Standardized scoring of cognitive assessments, such as by the use of Z-scores would also help with future aggregation of data. This review has identified few studies that take both noise and air pollution into account [28,50] and future studies need to consider both exposures, as evidence is emerging that air pollution may impact on cognitive functioning across the life course [59,60]: exposure to air pollution during the prenatal period has also been shown to impact on early childhood cognition [60]. We identified no studies of wind farm noise on children’s cognition.

## 5. Conclusions

In terms of environmental noise effects on cognition, this review has found that the quality of the evidence when considered across studies, ranges from being of moderate quality for an effect for some outcomes, e.g., reading comprehension, long-term memory, but is indicative of no substantial effect for other outcomes such as attention and executive function. These conclusions are limited by the low number of studies for some outcomes, and in particular for some environmental noise exposures such as road traffic noise and railway noise. The low quality evidence across studies for noise effects in some cognitive domains does not necessarily mean that there are no effects: rather, that more robust studies and a greater number of studies are required.

## Figures and Tables

**Table 1:** Summary of key features of studies on cognition.

	Number of Papers Out of 34	% of Papers Out of 34
**Noise Exposure**		
Road noise	11	32
Aircraft noise	25	74
Rail noise	3	9
Combined or ambient noise	3	9
Co-exposures, e.g., air pollution	2	6
**Noise Metric**		
L_Aeq_	27	79
L_dn_	4	12
% time above	3	9
L_Amax_ or no of events above L_Amax_	7	21
**Study Design**		
Cross-sectional	28	82
Longitudinal	7	21
Intervention	5	15
**Setting**		
School	30	88
Home	12	35
**Population**		
Children	34	100
Adults	0	0
**Outcome**		
Reading and oral comprehension	14	41
Short-term and long-term memory	12	35
Attention	13	38
Impairment assessed through standardized assessments such as SATs	13	38
Executive function deficit (working memory capacity, reasoning, task flexibility, problem solving)	9	26

**Table 2:** Summary of quality of the evidence and assessment of effect for environmental noise effects on cognition.

	Environmental Noise Exposure		
Cognitive Domain	Aircraft Noise: Quality of Evidence & Assessment of Effect	Road Traffic Noise: Quality of Evidence & Assessment of Effect	Railway Noise: Quality of Evidence & Assessment of Effect
Reading and oral comprehension	Moderate quality—harmful effect	Very low quality—no effect	n.a.
Standardized assessment tests	Moderate quality—harmful effect	Very low quality—harmful effect	Moderate quality—harmful effect
Long-term and short-term memory	Moderate quality—harmful effect	Very low quality—harmful effect	Very low quality—harmful effect
Attention	Low quality—no effect	Very low quality—no effect	Very low quality—no effect
Executive function	Very low quality—no effect	Low quality—no effect	n.a.

n.a. no studies available to evaluate.

**Table 3:** GRADE for the quality of evidence of environmental noise being associated with reading and oral comprehension.

	Aircraft Noise (14 Studies)			Road Traffic Noise (2 Studies)		
Domains	Criterion	Assessment	Downgrading	Criterion	Assessment	Downgrading
Start Level	Longitudinal or Intervention	4 Longitudinal Studies	High Quality	Cross-Sectional		Low Quality
1. Study Design	Majority of studies with low risk of bias	Low risk of bias	No downgrade	Majority of studies low quality	Low risk of bias	No downgrade
2. Inconsistency	Conflicting results; high I^2^	Inconsistent evidence; I^2^ not assessed	Downgrade	Conflicting results; high I^2^	Unable to assess consistency	Downgrade
3. Indirectness	Direct comparison; same PECCO	Did not make indirect comparison	No downgrade	Direct comparison; same PECCO	Did not make indirect comparison	No downgrade
4. Precision	Confidence interval contains 25% harm or benefit	Unable to rate for narrative review	No downgrade	Confidence interval contains 25% harm or benefit	Unable to rate for narrative review	No downgrade
5. Publication Bias	Funnel plot indicates	Suspected but unable to rate for narrative review	No downgrade	Funnel plot indicates	Suspected but unable to rate for narrative review	No downgrade
**Overall Judgement**			**Moderate Quality**			**Very low Quality**
6. Dose-response	Significant trend	Yes	No upgrade	Significant trend	Yes	No upgrade
7. Magnitude of effect	RR > 2	Not assessed	No upgrade	RR > 2	Not assessed	No upgrade
8. Confounding adjusted	Effect in spite of confounding working towards the nil	Good control for confounding	No upgrade	Effect in spite of confounding working towards the nil	Good control for confounding	No upgrade
**Overall Judgement**			**Moderate quality**			**Very low quality**

**Table 4:** GRADE for the quality of evidence of environmental noise associated with standardized assessment tests.

	Aircraft Noise (7 Studies)			Road Traffic Noise (4 Studies)			Railway Noise (2 Studies)		
Domains	Criterion	Assessment	Downgrading	Criterion	Assessment	Downgrading	Criterion	Assessment	Downgrading
Start Level	Intervention/Longitudinal	2 Longitudinal Studies	High Quality	Cross-Sectional		Low Quality	Intervention	1 Longitudinal Study	High Quality
1. Study Design	Majority of studies with low risk of bias	High risk of bias	Downgrade	Majority of studies low quality	High risk of bias	Downgrade	Majority of studies low quality	High risk of bias	Downgrade
2. Inconsistency	Conflicting results; high I^2^	Consistent evidence; I^2^ not assessed	No downgrade	Conflicting results; high I^2^	Consistent evidence; I^2^ not assessed	No downgrade	Conflicting results; high I^2^	Consistent evidence; I^2^ not assessed	No downgrade
3. Indirectness	Direct comparison; same PECCO	Did not make indirect comparison	No downgrade	Direct comparison; same PECCO	Did not make indirect comparison	No downgrade	Direct comparison; same PECCO	Did not make indirect comparison	No downgrade
4. Precision	Confidence interval contains 25% harm or benefit	Unable to rate for narrative review	No downgrade	Confidence interval contains 25% harm or benefit	Unable to rate for narrative review	No downgrade	Confidence interval contains 25% harm or benefit	Unable to rate for narrative review	No downgrade
5. Publication Bias	Funnel plot indicates	Suspected but unable to rate for narrative review	No downgrade	Funnel plot indicates	Suspected but unable to rate for narrative review	No downgrade	Funnel plot indicates	Suspected but unable to rate for narrative review	No downgrade
**Overall Judgement**			**Moderate quality**			**Very Low quality**			**Moderate quality**
6. Dose-response	Significant trend	Not assessed	No upgrade	Significant trend	Not assessed	No upgrade	Significant trend	Not assessed	No upgrade
7. Magnitude of effect	RR > 2	Not assessed	No upgrade	RR > 2	Not assessed	No upgrade	RR > 2	Not assessed	No upgrade
8. Confounding adjusted	Effect in spite of confounding working towards the nil	Omits control for individual level socioeconomic confounding	No upgrade	Effect in spite of confounding working towards the nil	Omits control for individual level socioeconomic confounding	No upgrade	Effect in spite of confounding working towards the nil	Omits control for individual level socioeconomic confounding	No upgrade
**Overall Judgement**			**Moderate quality**			**Low quality**			**Moderate quality**

**Table 5:** GRADE for the quality of evidence of environmental noise being associated with short-term and long-term memory.

	Aircraft Noise (11 Studies)			Road Traffic Noise (5 Studies)			Railway Noise (1 Study)		
Domains	Criterion	Assessment	Downgrading	Criterion	Assessment	Downgrading	Criterion	Assessment	Downgrading
Start Level	Intervention/Longitudinal	1 Longitudinal Study	High Quality	Cross-Sectional		Low Quality	Cross-Sectional		Low Quality
1. Study Design	Majority of studies with low risk of bias	Low risk of bias	No downgrade	Majority of studies low quality	Low risk of bias	No downgrade	Majority of studies low quality	Low risk of bias	No downgrade
2. Inconsistency	Conflicting results; high I^2^	I^2^ not assessed	Downgrade	Conflicting results; high I^2^	Inconsistent evidence; I^2^ not assessed	Downgrade	Conflicting results; high I^2^	I^2^ not assessed	Downgrade
3. Indirectness	Direct comparison; same PECCO	Did not make indirect comparison	No downgrade	Direct comparison; same PECCO	Did not make indirect comparison	No downgrade	Direct comparison; same PECCO	Did not make indirect comparison	No downgrade
4. Precision	Confidence interval contains 25% harm or benefit	Unable to rate for narrative review	No downgrade	Confidence interval contains 25% harm or benefit	Unable to rate for narrative review	No downgrade	Confidence interval contains 25% harm or benefit	Unable to rate for narrative review	No downgrade
5. Publication Bias	Funnel plot indicates	Suspected but unable to rate for narrative review	No downgrade	Funnel plot indicates	Suspected but unable to rate for narrative review	No downgrade	Funnel plot indicates	Suspected but unable to rate for narrative review	No downgrade
**Overall Judgement**			**Moderate quality**			**Very low quality**			**Very low quality**
6. Dose-response	Significant trend	Assessed in some studies but inconsistent findings	No upgrade	Significant trend	Assessed in some studies but inconsistent findings	No upgrade	Significant trend	Assessed in some studies but inconsistent findings	No upgrade
7. Magnitude of effect	RR > 2	Not assessed	No upgrade	RR > 2	Not assessed	No upgrade	RR > 2	Not assessed	No upgrade
8. Confounding adjusted	Effect in spite of confounding working towards the nil	Fine	No upgrade	Effect in spite of confounding working towards the nil	Fine	No upgrade	Effect in spite of confounding working towards the nil	Fine	No upgrade
**Overall Judgement**			**Moderate quality**			**Very low quality**			**Very low quality**

**Table 6:** GRADE for the quality of evidence of environmental noise being associated with attention.

	Aircraft Noise (10 Studies)			Road Traffic Noise (5 Studies)			Railway Noise (1 Study)		
Domains	Criterion	Assessment	Downgrading	Criterion	Assessment	Downgrading	Criterion	Assessment	Downgrading
Start Level	Longitudinal/Intervention	1 Intervention and 2 Longitudinal Studies	High Quality	Cross-Sectional		Low Quality	Cross-Sectional		Low Quality
1. Study Design	Majority of studies with low risk of bias	High risk of bias	Downgrade	Majority of studies low quality	High risk of bias	Downgrade	Majority of studies low quality	Low risk of bias	No downgrade
2. Inconsistency	Conflicting results; high I^2^	Inconsistent evidence; I^2^ not assessed	Downgrade	Conflicting results; high I^2^	Inconsistent evidence; I^2^ not assessed	Downgrade	Conflicting results; high I^2^	I^2^ not assessed	Downgrade
3. Indirectness	Direct comparison; same PECCO	Indirect comparison	No downgrade	Direct comparison; same PECCO	Indirect comparison	No downgrade	Direct comparison; same PECCO	Did not make indirect comparison	No downgrade
4. Precision	Confidence interval contains 25% harm or benefit	Unable to rate for narrative review	No downgrade	Confidence interval contains 25% harm or benefit	Unable to rate for narrative review	No downgrade	Confidence interval contains 25% harm or benefit	Unable to rate for narrative review	No downgrade
5. Publication Bias	Funnel plot indicates	Suspected but unable to rate for narrative review	No downgrade	Funnel plot indicates	Suspected but unable to rate for narrative review	No downgrade	Funnel plot indicates	Suspected but unable to rate for narrative review	No downgrade
**Overall Judgement**			**Low Quality**			**Very low Quality**			**Very low quality**
6. Dose-response	Significant trend	Examined in limited number of studies	No upgrade	Significant trend	Examined in limited number of studies	No upgrade	Significant trend	Assessed in some studies but inconsistent findings	No upgrade
7. Magnitude of effect	RR > 2	Not assessed	No upgrade	RR > 2	Not assessed	No upgrade	RR > 2	Not assessed	No upgrade
8. Confounding adjusted	Effect in spite of confounding working towards the nil	Fine	No upgrade	Effect in spite of confounding working towards the nil	Fine	No upgrade	Effect in spite of confounding working towards the nil	Fine	No upgrade
**Overall Judgement**			**Low quality**			**Very low quality**			**Very low quality**

**Table 7:** GRADE for the quality of evidence of environmental noise being associated with executive function.

	Aircraft Noise (9 Studies)			Road Traffic Noise (5 Studies)		
Domains	Criterion	Assessment	Downgrading	Criterion	Assessment	Downgrading
Start Level	Cross-Sectional		Low Quality	Cross-Sectional		Low Quality
1. Study Design	Majority of studies with low risk of bias	Low risk of bias	No downgrade	Majority of studies low quality	Low risk of bias	No downgrade
2. Inconsistency	Conflicting results; high I^2^	Consistent; I^2^ not assessed	Downgrade	Conflicting results; high I^2^	Consistent; I^2^ not assessed	No downgrade
3. Indirectness	Direct comparison; same PECCO	Indirect comparison	No downgrade	Direct comparison; same PECCO	Indirect comparison	No downgrade
4. Precision	Confidence interval contains 25% harm or benefit	Unable to rate for narrative review	No downgrade	Confidence interval contains 25% harm or benefit	Unable to rate for narrative review	No downgrade
5. Publication Bias	Funnel plot indicates	Suspected but unable to rate for narrative review	No downgrade	Funnel plot indicates	Suspected but unable to rate for narrative review	No downgrade
**Overall Judgement**			**Very low quality**			**Low quality**
6. Dose-response	Significant trend	Not demonstrated	No upgrade	Significant trend	Not demonstrated	No upgrade
7. Magnitude of effect	RR > 2	Not assessed	No upgrade	RR > 2	Not assessed	No upgrade
8. Confounding adjusted	Effect in spite of confounding working towards the nil	Fine	No upgrade	Effect in spite of confounding working towards the nil	Fine	No upgrade
**Overall Judgement**			**Very low quality**			**Low quality**

## Supplementary Materials

The following are available online at http://www.mdpi.com/1660-4601/15/2/285/s1, Table S1: Characteristics of included studies for environmental noise effects on reading and oral comprehension, Table S2: Characteristics of included studies for environmental noise effects on standardized assessment test scores, Table S3: Characteristics of included studies for environmental §noise effects on long-term & short-term memory, Table S4: Characteristics of included studies for environmental noise effects on attention, Table S5: Characteristics of included studies for environmental noise effects on executive function deficit (working memory).

## Appendix A

Web Appendix: Search string

Search Terms: (note wind noise terms were included in initial search for systematic reviews but not in second search for individual papers)

environmental noise; community noise; traffic noise; wind turbine noise; wind farm noise; wind turbine sound; wind farm sound; aircraft noise; airport noise; railway noise; road traffic noise; transportation noise; train noise; leisure noise; leisure-time noise; neighbourhood noise; neighborhood noise; household noise; low frequency noise; classroom noise; school noise; high-volume music; high-volume noise; noise from personal electronic devices; noise from mp3 players; noise from children’s toys; hospital noise; combined noise exposure; noise nuisance; noise exposure; truck noise; motor vehicle noise; noise load; entertainment noise; noise from mobile phones; noise from personal audio devices; noise from personal music players; combined exposure to noise and vibration; combined exposure to noise and air pollution; prospective; retrospective; cohort studies; case-control; observational; experimental; cross-sectional; learning impairment; reading and oral comprehension; short-term memory; long-term memory; attention; impairment assessed through standardized assessments; children; cognitive impairment in the elderly and working age population: concentration; speech intelligibility; executive function deficit; working memory; memory capacity; reasoning; task flexibility; problem solving; hyperactivity.
