# Supplementary material for: WHO Environmental Noise Guidelines for the European Region: A Systematic Review on Environmental Noise and Cognition
Source: Int J Environ Res Public Health. 2018 Feb 7;15(2):285. doi: 10.3390/ijerph15020285 (PMC5858354; doi:10.3390/ijerph15020285)
Supplement: Supplementary file 1 [file ijerph-15-00285-s001.pdf]

Table S1: Characteristics of included studies for environmental noise effects on reading and oral comprehension

	Reference	**Population**: general population in settings (hospitals, residences, public venues, educational facilities) + **response rate** and other **selection** /bias factors **Cross-sectional or longitudinal**	**Exposure**: exposure to high levels of environmental noise from various noise sources + noise metric involved + modelled or measured noise	**Comparator**: no noise exposure or lower levels of noise exposure	**Confounding**: adjusted for confounding	**Outcome**: assessment of outcome	**Findings:** expressed as effect per dB if possible.<br><br>Type of analyses<br><br>Sample size relating to the effect size	**Comments:** Anything else to note
**AIRCRAFT NOISE EXPOSURE**								
**Intervention Evidence**								
1.	Hygge et al, Psychol Sci, 2002	**Population**: school children, noisy and quiet schools around the old and the new airport, total n=326, aged 8-12yrs. Four groups: n=43 old-airport, no-noise; n=65 old-airport plus noise; n=107 new-airport, no-noise; n=111 new-airport plus noise	**Noise exposure**: aircraft noise levels **measured** around schools before and after relocation of the airport; **Noise source**: aircraft noise **Noise metrics**: Leq, 24h (dBA)	**Comparison**: children from noisy schools around the old and the new airport compared to children from quiet schools around the old and the new airport	**Confounding**: None	**Outcomes:** **Reading test** in German (Biglmaier, 1969), **Long-term memory** – recall of a text read in noisy conditions the previous day, **Short-term memory** – remembering consonants), **Attention** – visual search and reaction time were	**Findings:** **At the new airport** children showed a decrease of the number of correct answers on long-term memory task, and impairment of speech perception. **At the old airport** children showed decrease in the number of errors on reading test,	**Comments:** **Findings:** The effect of noise on the reading tasks was not mediated by memory or speech perception. Poorer reading was not mediated by speech perception, and impaired recall was in part mediated by

		**Inclusion criteria**: 2 years of residence, speaking German fluently, normal hearing (assessed by audiometric screening) **Longitudinal study;** baseline (wave 1): 6 months before the opening of new airport, follow-up 1 and 2 years (waves 2 and 3) after changeover of airports **Intervention Study**				tested in both quiet and noisy conditions, **Speech perception** – hearing a story against noisy background (adapted from Hygge, Rönnberg, Larsby, Arlinger, 1992)	increase of the number of correct answers on short-term memory task, and improvement of speech perception. **Type of analyses:** t-test for independent samples, multivariate analyses of variance MANOVA **Sample size relating to the effect size:** n=326	reading.
2.	Seabi et al, J Exp Science Eviron Epidem, 2013	**Population**: school children, before and after the relocation of an international airport, aged 9-15yrs. **Sampling procedure**: first testing in 2009: 732 children aged 11yrs; second testing,	**Noise exposure**: noise levels **measured** outside schools from 8 am to 10:30 am **Noise groups**: high noise group – schools near flight path in 2009, but after relocation of airport noise level was reduced (2010 and 2011 testing)	**Compared to:** low noise group – schools in quiet areas in 2009; remained quiet in 2010 and 2011	**Confounding**: Age, gender, home language (English first language, English second language), socio-economic deprivation (eligible for free meal at school)	**Outcome**: **Reading comprehension** – tested using Suffolk Reading Scale Level 2 (SRS2) (Hagley, 2002)	**Findings:** No difference in reading conmprehention scores between low noise and high noise groups after relocation of airport; the increase of reading comprehension over time was not	**Comments:** **Noise exposure:** Noise levels measured in 2010 and 2011 in high noise and low noise groups were similar (because of the removal of airport from high noise area in 2010).

		2010: 650 children aged 12.3yrs; third testing, 2011: 178 children aged 13.1yrs **Longitudinal study;** follow-up period: 3 years (2009-2011)	**Noise source**: aircraft predominant, other source was road traffic **Noise metrics:** Leq (dBA)				related to groups or time; the removal of **aircraft** noise did not lead to improved reading comprehension **Type of analyses**: repeated multiple analyses of covariance (MANCOVA) **Sample size relating to the effect size:** n=93 from high noise group and n=85 from low noise group	**Findings**: Significant confounding role of home language on reading comprehension. Increase of reading comprehension in both groups over time.
**Longitudinal Evidence**								
3.	Clark et al, J Enviro Psychol, 2013	**Population**: school children, total n=461, aged 15-16yrs. **Sampling procedure**: baseline sample tested in 2001-2003: 1355 children aged 9-10yrs; follow-up sample testing in 2008: 1015 children eligible for testing, 461 children	**Noise exposure**: aircraft noise levels at primary and secondary schools **measured** in an area from 7 am to 11 pm; road traffic noise at school **combined** from measurements and models available for elementary schools **Noise source**:	**Compared to**: noise levels at secondary schools compared to noise levels at primary schools	**Confounding**: age, gender, employment, home ownership, home crowding, mother's educational attainment, long-standing illness, parental support for school work at baseline, classroom glazing at primary school	**Outcome**: **Reading comprehension** – Suffolk Reading Scale (SRS2) Level 2 and Level 3 (Hagley, 2002)	**Findings:** Increase of **aircraft** noise at secondary school by 1 dB was non-significantly associated with a decrease of the performance on reading test by 0.022 marks (unadjusted), or 0.016 marks (adjusted) **Type of analyses:** Multilevel linear	**Comments:** **Findings:** For the majority of children noise exposure levels at primary and secondary schools were similar, some children moved from quieter to noisier schools, and vice versa.

		participated, aged 15-16; response rate 45.4%<br>**Longitudinal study;** follow-up period: 6 years (2001/2003-2008)	aircraft and road traffic<br>**Noise metrics**: LAeq, 16h (dBA)				regression analyses<br>**Sample size relating to the effect size:**<br>n=461 with complete data	
	See Hygge et al, Psychol Sci, 2002 above							
4.	Haines et al, Int J Epidemiol, 2001	**Population**: school children, noisy and quiet schools around the airport, total n=275, aged 8-11yrs.<br>**Sampling procedure**: baseline sample tested in 1996: n=340 children; follow-up sample tested in 1997: response rate 81%, n=275<br>**Longitudinal study** at two time points	**Noise exposure**: aircraft noise levels **modelled** around schools; acute noise exposure measured inside classrooms<br>**Noise source**: aircraft noise<br>**Noise metrics**: LAeq, 16h (dBA)<br>**Noise groups**: High-aircraft noise-impact schools (Leq, 16h >66 dBA), low-aircraft noise-impact schools (Leq, 16h <57 dBA)	**Comparison**: children from noisy schools around compared to children from quiet schools	**Confounding**: Age, household deprivation score – incorporating income, home ownership, unemployment, adapted from Townsend's Scale (Townsend, et al, 1989), main language spoken at home	**Outcome**:<br>**Reading comprehension** – Suffolk Reading Scale (SRS2) Level 2 and Level 3 (Hagley, 2002)<br>**Sustained Attention** – score task from Tests of Everyday Attention for Children (TEA-Ch) (Manly et al, 1998)	**Findings:** Children from high-**aircraft** noise schools had poorer reading comprehension and poorer sustained attention.<br>**Type of analyses:** Analyses of covariance ANCOVA<br>**Sample size relating to the effect size:**<br>n=148 from high-aircraft noise exposed schools, n=127 from low-aircraft noise exposed schools	**Comments:** At follow-up, reading comprehension was poorer among children from high-aircraft noise schools, but non-significant after adjustment. This implies that the effect of noise on reading comprehension may be influenced by socio-demographic factors.
	See Seabi et al, J Exp Science							

	Eviron Epidem, 2013 above							
**Cross-sectional Studies**								
5.	Clark et al, Am J Epidemiol, 2006	**Population**: school children, 89 schools around three airports, total n=2010, aged 9-10yrs. **Sampling**: 3207 children approached; 2844 pupils participated; response rate: 89% of children, 80% of parents **Cross sectional** study	**Noise exposure**: aircraft noise levels at home and at school **measured** in an area from 7 am to 11 pm; road traffic noise at school **modelled** or **combination** of measurements with models **Noise source**: aircraft and road traffic **Noise metrics**: LAeq, 16h (dBA)	**Comparator**: lower levels of noise exposure	**Confounding**: age, gender, country, mother's education, parental employment status, home crowding, home ownership, long-standing illness, main language spoken at home, parental support for schoolwork, classroom glazing; dyslexia, hearing impairment, noise during testing; aircraft noise annoyance, cognitive outcomes, country	**Outcome**: **Reading comprehension** Suffolk Reading Scale Level 2 (SRS2) (Hagley, 2002), CITO Readibility Index for elementary and Special Education (Staphorsius, 1994), Ecaluacion Comprension Lectora ECL-2 (De La Cruz, 1999); **Episodic memory** – Child Memory scale, (Cohen, 1997); **Sustained attention** – Toulouse Pieron test (Toulouse et al, 1986); **Working memory** – Search and Memory test (Smith and Miles, 1987; Hygge et al, 2003)	**Findings:** Increase of **aircraft** noise at school by 1 dBA correlated to decrease of the performance on the reading test by 0.008 marks; Increase of **aircraft** noise at home by 1 dBA correlated to decrease of the performance on the reading test by 0.008 marks **Type of analyses:** multilevel model analyses (for data clustering) **Sample size relating to the effect size:** n=2010 with complete data	**Comments: Findings:** Significant correlation between aircraft noise at home and at school; No correlation between road traffic noise at school and reading comprehension; findings consistent across all three countries
6.	Clark et al, Am J Epidemol, 2012	**Population**: school children, total n=960, aged 9-10yrs.	**Noise exposure**: aircraft noise levels at home and at school	**Comparator**: lower levels of noise exposure	**Confounding**: Modelled concentrations of **NO2** (µg/m3);	**Outcome**: **Reading comprehension** Suffolk Reading	**Findings:** Increase of **aircraft** noise at school by 1 dBA	**Comments: Findings:** Moderate correlation

		**Cross sectional** study	**measured** in an area from 7 am to 11 pm; road traffic noise at school **combined** from measurements and models **Noise source**: aircraft and road traffic **Noise metrics**: LAeq, 16h (dBA)		Socio-economic status (employment, housing tenure, home crowding), maternal education, ethnicity, main language spoken at home; age, gender, long-standing illness, parental support for schoolwork, classroom glazing; other noise exposure source	Scale Level 2 (SRS2) (Hagley, 2002), CITO Readibility Index for elementary and Special Education (Staphorsius, 1994), Ecaluacion Comprension Lectora ECL-2 (De La Cruz, 1999); **Episodic memory** tested with Child Memory scale, (Cohen, 1997)	correlated to decrease of the performance on the following tests: reading comprehension (by 0.01 marks), recognition memory (by 0.045 marks), information recall (by 0.043 marks) and conceptual recall (by 0.015 marks). **Type of analyses:** Multilevel linear and logistic regression models **Sample size relating to the effect size:** n=719 with air pollution data and n=241 without air pollution data	between NO2 levels and aircraft noise and road traffic noise. No association between road traffic noise and reading comprehension
7.	Haines, Stansfeld, Brentnall et al, Psychol Med, 2001	**Population**: school children, 20 schools, total n=451, aged 8-11yrs. Noisy and quiet schools matched for age, sex, other noise sources, noise protection at	**Noise exposure**: aircraft noise levels **modelled** around schools **Noise source**: aircraft noise **Noise metrics**: LAeq, 16h (dBA) **Noise groups**: noisy schools (Leq,	**Comparator**: Children from high aircraft noise schools compared with children from lower aircraft noise level schools	**Confounding**: Household deprivation score – incorporating income, home tenure, car ownership, employment, central heating, social class and	**Outcome**: **Reading comprehension** – Suffolk Reading Scale (SRS2) Level 2 (Hagley, 2002); **Long-term memory** – including immediate recall,	**Findings:** Children in high noise schools had poor performance on the difficult items (20% of all items) of the reading scale. **Type of analyses:** Analysis of	**Comments:** **Findings:** No difference in reading, immediate recall, delayed recall, recognition memory, sustained attention and

		school, socio-economic status, main language; Response rate 82% **Cross-sectional study**	16h >63 dBA), quiet schools (Leq, 16h <57 dBA)		household crowding (Townsend, et al, 1989), age, main language spoken at home	delayed recall, and recognition – adapted from Child Memory Scale (Cohen, 1997); **Backward serial digit recall** (Pickering and Gathercole, 2000); **Sustained Attention** – score task from Tests of Everyday Attention for Children (TEA-Ch) (Manly et al, 1998)	covariance (ANCOVA), Multilevel modeling analysis **Sample size relating to the effect size:** n=451 (n=236 from high-level noise schools, n=215 from low-level noise schools)	serial backward digit recall between children from high-level aircraft noise schools and children from low level noise schools.
8.	Haines, Stansfeld, Job et al, Psychol Med, 2001	**Population**: school children, 8 schools, total n=340, aged 8-11yrs. Noisy and quiet schools matched for age, sex, other noise sources, noise protection at school, socio-economic status, ethnic groups; Response rate 77% **Cross-sectional study**	**Noise exposure**: aircraft noise levels **modelled** around schools; acute noise levels **measured** indoors **Noise source**: aircraft noise **Noise metrics**: LAeq, 16h (dBA) outdoors; single event noise exposure level (SEL dBA); LAeq during time needed for task completion **Noise groups**: noisy schools (Leq,	**Comparator**: Children from high aircraft noise schools compared with children from lower aircraft noise level schools	**Confounding**: Household deprivation score – incorporating income, crowding, home ownership, unemployment, (Townsend, et al, 1989), age, main language spoken at home	**Outcome**: **Reading comprehension** – Suffolk Reading Scale (SRS2) Level 2 (Hagley, 2002); **Long-term memory** – including recognition task and recall task (Evans et al, 1995); **Short-term memory** – serial digit recall task; **Motivation – performance measure**, i.e. solving insolvable	**Findings:** Children in high noise schools had poor performance on the reading scale than children from low noise schools (even after adjustment for deprivation, age, language). Children in high noise schools had poor long-term memory than children from low noise schools (only a subsample	**Comments:** **Findings:** Acute aircraft noise exposure at the time of testing was not associated with reading comprehension or annoyance. **Conclusion:** The association between aircraft noise exposure and reading comprehension is independent from noise annoyance, acute

			16h >66 dBA), quiet schools (Leq, 16h <57 dBA)			and solvable puzzle (Evans et al, 1995); **Child Attributional Style** – measured by Child Attribution Style Questionnaire (CASQ; Kaslow & Nolen-Hoeksema, 1991); **Classroom motivation, i.e. learned helplessness** – estimated by teachers using Student Behaviour Checklist (SBC; Fincham et al. 1989)	without bias). No effect of noise exposure on motivation, self-reported attributional scale and classroom motivation. **Type of analyses:** Analysis of covariance (ANCOVA) **Sample size relating to the effect size:** n=340 (n=169 from high-level noise schools, n=171 from low-level noise schools)	noise levels, age, language, and deprivation.
9.	Klatte et al, Report, NORAH study, 2014	**Population**: 85 second-grade classes from 29 primary schools around Frankfurt/Main airport; children: total n=1243, age 8yrs, 4months **Cross-sectional** study	**Noise exposure:** Noise contours at school and at home around airport **Noise source:** aircraft noise **Noise metrics:** Leq around schools (time 8-14h); average 49.5 dB, Median 50.6 dB; range 39 to 59 dB; Leq at home (6-18h)	**Comparison:** Change estimate per 10 dBA increase	**Confounding:** Migrant background. Children´s exposure to road traffic and railway noise. Also used as confounders: auditory thinking, phonological awareness, episodic memory. Class characteristics (size, proportion	**Outcomes:** **Reading test**: standardized comprehension test for primary school children (ELFE 1-6, Lenhard & Schneider, 2006). **Language functions** – speech perception, auditory thinking, phonological short-term	**Findings:** A 10 dB increase in aircraft noise associated with a decrement of one-tenth of an SD on the **reading** test, corresponding to a one month reading delay in this test. Similar results for subscales: word and text	**Comments:** **Methods:** Impossible to separate home and schools exposure (high correlation). **Findings:** Significant confounding of migrant background – aircraft noise (both home and school) strongly

			**Noise groups:** 3 groups at school: low exposure < 47 dB; middle exposure 47 to < 55 dB; high exposure ≥ 55 dB		of migrants, socioeconomic status, parental engagement), classroom reverberation and insulation.	memory, phonological awareness, episodic memory test. **Other outcomes**: Health-related quality of life, well-being at home and school, noise annoyance at home and school.	comprehension. Similar results found for aircraft noise at home. **Type of analyses:** Multilevel regression **Sample size relating to the effect size:** n=1090	affects **reading** comprehension of children without migration background (1.5-2.5 months reading delay). No effect of aircraft noise on tested **language** functions. Aircraft noise at school and at home significantly associated with **annoyance**. Aircraft noise was associated with less positive judgments of health-related **quality of life**, well-being at school, and sleep quality.
10.	Matsui et al, Noise Health, 2004	**Population**: school children, high aircraft noise exposure at school, three groups of aircraft noise exposure at home; total n=451, aged 8-	**Noise exposure**: aircraft noise levels at home and at school **modelled** **Noise source**: aircraft noise **Noise metrics**: LAeq, 16h (dB)	**Comparator**: children from homes with low noise levels (LAeq, 16h < 63 dB) compared to children from schools with	**Confounding:** Age, sex, schools, household deprivation score (including income, home tenure, car ownership, employment, central heating,	**Outcome**: **Reading comprehension** Suffolk Reading Scale Level 2 (Hagley, 1987); **Long term Memory Recall and Recognition** –	**Findings:** **Aircraft** noise levels at home significantly predict poor performance at immediate recall and delayed recall tests.	**Findings:** No association between aircraft noise levels at home and delayed recognition, sustained attention and

		9yrs, response rate: 83% in high noise schools and 81% in low noise schools **Cross sectional** study	**Noise groups**: All children attending schools with high levels of aircraft noise (LAeq, 16h > 63 dB), but with different levels of aircraft noise at home (LAeq, 16h < 63dB vs. LAeq, 16h =63-66 dB, vs. LAeq, 16h > 63 dB)	higher noise levels (LAeq, 16h =63-66 dB), and to the highest noise levels (LAeq, 16h > 63 dB)	household crowding, social class), mother's educational level, long-standing illness, main language spoken at home	tested with Children's Memory scale (Cohen, 1997); **Sustained Attention** tested with Tests of Everyday Attention for Children (TEA-Ch) (Manly et al, 1998)	**Type of analyses:** Multiple logistic regression analyses **Sample size relating to the effect size:** n=163 with complete data	reading comprehension.
11.	Seabi et al, Noise Health, 2012	**Population**: school children from five schools, 834 children approached, sample n=693, aged 9-14yrs. **Inclusion criteria**: minimum of 2 yrs of residence in the study area, normal hearing (perceived by parents or teachers), being in grade 5 or 6 **Exclusion criteria**: learning difficulties, auditory processing	**Noise exposure**: noise levels **measured** outside schools from 8 am to 10:00 am **Noise groups**: high noise group – schools near aircraft flight path **Noise source**: aircraft noise **Noise metrics:** Leq (dBA)	**Compared to:** low noise group – schools in quiet urban areas	**Confounding**: Age, gender, race, home language (English first language, English second language), socio-economic deprivation (eligible for free meal at school), intellectual ability (tested with the Figure Analogies subtest of the Quantitative Battery for Cognitive Abilities Test (Lohman et al, 2001))	**Outcome**: **Reading comprehension** – tested using Suffolk Reading Scale Level 2 (SRS2) (Hagley, 2002)	**Findings:** Children from high **aircraft** noise groups had lower reading comprehension scores compared to low noise group. **Type of analyses**: Not mentioned in the paper; possibly MANCOVA; univariate general linear model **Sample size relating to the effect size:** n=313 from high noise group and n=380 from low noise group	**Comments: Findings**: Children whose first language is English had higher reading comprehension scores; the effect remained stable after adjusting for gender, intellectual ability and socio-economic status; significant interaction between noise and primary language spoken.

		disorders and/or attentional problems **Cross sectional study**						
12.	Stansfeld et al, Lancet, 2005	**Population**: school children, 89 schools around three airports, total n=2844, aged 9-13yrs. **Cross sectional** study	**Noise exposure**: aircraft noise levels at home and at school **measured** in an area from 7 am to 11 pm; road traffic noise at school **modelled** or **combination** of measurements with models **Noise source**: aircraft and road traffic **Noise metrics**: LAeq, 16h (dBA)	**Comparator**: lower levels of noise exposure	**Confounding**: age, gender, parental employment, home ownership, home crowding, mother's educational attainment, long-standing illness, main language spoken at home, parental support for school work, classroom glazing	**Outcome**: **Reading comprehension** Suffolk Reading Scale Level 2 (SRS2) (Hagley, 2002), CITO Readibility Index for elementary and Special Education (Staphorsius, 1994), Ecaluacion Comprension Lectora ECL-2 (De La Cruz, 1999); **Episodic memory** – Child Memory scale, (Cohen, 1997); **Sustained attention** – Toulouse Pieron test (Toulouse et al, 1986); **Working memory** – Search and Memory test (Smith and Miles, 1987; Hygge et al, 2003); **Prospective memory** (writing	**Findings:** Increase of **aircraft** noise at school by 1 dBA correlated to decrease of the performance on the reading test by 0.008 marks; Increase of aircraft noise at school by 1 dBA correlated to decrease of the performance on the recognition test by 0.018 marks. Increase of **road traffic** noise by 1 dB was associated with an increase of information recall by 0.038 marks and an increase of conceptual recall by 0.013 marks. **Type of analyses:** Multilevel model	**Comments:** **Findings:** No association between aircraft and road traffic noise and cued conceptual recall, cued information recall, prospective memory, working memory, and sustained attention.

						initials as instructed)	analyses (for data clustering)<br>**Sample size relating to the effect size:** range from 1939 to 2014 with complete data	
13.	Stansfeld, Hygge, Clark et al, Noise Health, 2010	**Population**: school children, two studies re-analysed.<br>**Munich study**: n=326, aged 9-11yrs, around old and new airport<br>Inclusion criteria: 2 years of residence, speaking German fluently<br>**Longitudinal study;** baseline (wave 1): 6 months before the opening of new airport, follow-up 1 and 2 years (waves 2 and 3) after changeover of airports<br>**RANCH study:** n=857, aged 9-10yrs, around	**Noise exposure**: aircraft noise levels **measured** around schools in Munich study; **modeled** in RANCH study<br>**Noise source**: aircraft noise<br>**Noise metrics**: Munich study: Leq, 24h (dBA);<br>RANCH study: Leq, nighttime at home (dBA) (11 p.m. to 7 a.m.)	**Comparison**: Munich study: children from noisy schools around the old and the new airport compared to children from quiet schools around the old and the new airport.<br>RANCH study: higher levels of aircraft noise exposure compared to lower levels of noise	**Confounding**: Munich study: self-reported sleep quality; RANCH study: daytime aircraft noise at school, road-traffic noise, sleep problems, age, sex, parental employment, crowding, homeownership, mother's education, child's illness, main language at home, parental support for home work, classroom glazing	**Outcomes:**<br>**Munich study: Reading test** in German (Biglmaier, 1969) in noisy conditions (80 dBA)<br>**Long-term memory** – recall of a text read in noisy conditions the previous day.<br>**RANCH study: Reading comprehension** Suffolk Reading Scale Level 2 (SRS2) (Hagley, 2002),<br>**Episodic memory** – Child Memory Scale, (Cohen, 1997); **Sustained attention** – Toulouse Pieron test (Toulouse et al, 1986);	**Findings:**<br>**Munich study:** No association between **nighttime aircraft** noise and cognitive impairment (mediated by sleep quality).<br>**RANCH study:** Increase of **nighttime aircraft** noise at home by 1 dBA correlated to decrease of the reading test performance by 0.009 marks, and to decrease of recognition memory by 0.031 marks.<br>**Type of analyses:** Multilevel modeling<br>**Sample size relating to the**	**Comments:**<br>**Findings:**<br>RANCH study: Daytime and nighttime noise exposure highly correlated.<br>Nighttime aircraft exposure was no longer associated with impaired reading comprehension and recognition memory after adjustment for daytime aircraft noise at school.

		the airport **Cross-sectional** study				**Working memory** – Search and Memory test (Smith and Miles, 1987; Hygge et al, 2003)	**effect size:** n=326 (Munich study), n=842 for RANCH study	
14.	Evans and Maxwell, Enviro Behav, 1997	**Population**: school children, two schools of similar socio-economic characteristics, language and ethnicity, total n=116, aged 5-7yrs. **Cross-sectional** study	**Noise exposure**: flight contours **modeled** outside schools **Noise source**: aircraft noise **Noise metrics**: LAeq (dBA) **Noise groups**: high-noise school (within 65 dBA flight contour) vs. quiet school	**Comparison**: Children from a high-noise school compared to children from a quiet school	**Confounding:** Mother's education, income	**Outcomes:** **Reading skills** – Woodcock Reading Mastery Test (Woodcock, 1987); **Speech perception** – exposure to noise-masked words (Carrol et al, 1971); **Sound perception** – exposure to noise-masked environmental sounds (Brady et al, 1983); **Embedded phoneme test** – perception of sounf within different words (Fowler, 1990)	**Findings:** Chronic exposure to aircraft noise was inversely correlated with reading scores and speech perception. Noise was significant predictor of low reading scores, after adjustment for mother's education. **Type of analyses:** Linear regression, correlation **Sample size relating to the effect size:** n=116	**Comments:** **Findings:** Speech perception was a mediator between noise exposure and poor reading skills.
**ROAD TRAFFIC NOISE EXPOSURE**								
**Cross-sectional Evidence**								
	See Stansfeld et al, Lancet, 2005 above							
	See Clark et al, Am J Epidemiol, 2006							

Table S2: Characteristics of included studies for environmental noise effects on standardized assessment test scores

	Reference	**Population**: general population in settings (hospitals, residences, public venues, educational facilities) + **response rate** and other **selection** /bias factors **Cross-sectional or longitudinal**	**Exposure**: exposure to high levels of environmental noise from various noise sources + noise metric involved + modelled or measured noise	**Comparator**: no noise exposure or lower levels of noise exposure	**Confounding**: adjusted for confounding	**Outcome**: assessment of outcome	**Findings:** expressed as effect per dB if possible.<br><br>Type of analyses<br><br>Sample size relating to the effect size	**Comments:** Anything else to note

**Aircraft Noise Exposure**
**Intervention Evidence**

1.	Eagan et al, , 2004	**Population**: school children, 35 schools around 3 airports, total n=unspecified, aged 6-18yrs. **Selection:** schools that experienced reduction in noise levels (due to commercial airport closure or due to school sound insulation)<br><br>**Cross-sectional study Intervention** study	**Noise exposure**: noise levels **modeled** for school year, school months, and school hours; converted to indoor noise **Noise source**: aircraft noise **Noise metrics**: LAeq, school day (7 a.m. to 4 p.m.); speech interference level (SIL), including percent of time LAeq>40 dBA; number of events with LAmax >40 dBA; speech intelligibility index (SII) – number of events disrupting speech	**Comparison:** Change in test scores compared to change in noise reduction	**Confounding:** Prior test score, prior noise exposure, cause of airport's noise reduction combined with testing state, test-regime change in one state; age groups (elementary, middle, and high school); student group (individualized education program IEP and non-IEP)	**Outcomes: Standardized tests** – scores on two mandatory tests – verbal and math/science (average score, failure rate, i.e. % children with lowest score, top-score rate – i.e. % children with the best score)	**Findings:** Noise reduction was associated with a decrease in failure rate in high school pupils, but not in middle-school and elementary school pupils. Reduction in percent of time LAeq>40 dBA by 5% points was associated with reduction of failure rate in high school pupils by 20% points, and with reduction of top-score rate in children with disabilities (IEP) by 5% points. **Type of analyses:** Multi-variate multilevel regression **Sample size relating to the effect size:** n=not specified	**Comments: Methods:** Intervention / noise abatement study **Findings:** Reduction in percent of time LAeq>40 dBA by 5% points was associated with improvement of average scores by 7-9% points. Reduction in the number of events with LAmax>40 dBA by 20 events was associated with improvement of average scores by 4-5% points in elementary school pupils, but with a decrease of average scores by 17-19% points in high school pupils.

2.	FICAN et al, 2007 – see Eagan et al, 2004 above.							

3.	Sharp et al, Report, ACRP Document, 2014	**Population**: 6198 schools around 46 airports, data collected 2000-2008; children: total n=unspecified, age unspecified, 3rd, 4th, 5th grade<br>**Cross-sectional** study and **Intervention study** – examined effects after sub-sample of schools had sound insulation installed.	**Noise exposure**: noise levels **modeled** for school year, school months, and school hours<br>**Noise source**: aircraft noise<br>**Noise metrics**: fro ambient noise: Ldn; for school noise at school hours (7a.m. – 3p.m.): Lmax, SEL, Leq, time above a threshold noise level (TA), number of events above a threshold noise level (NA)<br>**Noise groups**: target schools (Ldn ≥55 dBA; n=917) vs. control schools (not exposed to aircraft noise) vs. insulated schools (n=173)	**Comparison:** Change estimate per 10 dBA increase	**Confounding:** Eligible for free meal, school's enrollment of children from minority groups, pupil-teacher ratio, average enrollment per grade in the school	**Outcomes:**<br>**School average test scores** on reading and mathematics; converted to index.<br>(Scores are not comparable between states, but comparable across schools in the same state and year). Scores were further adjusted and normalized to allow between-state comparisons – percentile of state ranking.	**Cross-sectional findings:**<br>An increase in **aircraft** noise by 10 dBA was related to a decrease in state ranking of a school by 1 percentile. An increase in ambient noise by 10 dBA was related to a decrease in state ranking of a school by 3 percentiles.<br>An increase in incremental noise by 10 dBA was related to a decrease in state ranking of a school by 6 percentiles.<br>**Intervention findings**: In a sub-sample of 119 schools, the effect of aircraft noise on children's learning disappeared once the school had sound insulation installed.<br><br>**Type of analyses:** Multilevel regression, GLM procedure<br>**Sample size relating to the effect size:** n=not specified	**Comments:**<br>**Method:** Comparison between schools, not between children.<br>**Findings:** When aircraft noise is 5 dBA greater than ambient noise, the percentile decrease in state ranking (of a school) is 3%.<br>The effect of aircraft noise was greater for non-disadvantaged children than for disadvantaged children.

Longitudinal Evidence

4.	Cohen et l, J Person Social Psychol, 1981<br><br>Cohen et al, American Scientist, 1981.	**Population**: school children, noisy and quiet schools around the airport, first testing n=262, second testing n=163, age unknown (third and fourth grade, possibly 9-10yrs)<br>**Sampling procedure:** Noisy and quiet schools matched for age, grade, ethnic and racial distribution, families with assistance, parental educational and occupational level<br>**Exclusion criterion**: hearing impairment (audiometric screening)<br>**Longitudinal study;** follow-up period: 1 year (1977-1978)<br>**Cross-sectional study:** comparison between noisy, noise-abated and quiet classrooms	**Noise exposure**: aircraft noise levels **measured inside** the classroom for 1 hour in the morning and in the afternoon<br>**Noise source**: aircraft noise<br>**Noise metrics**: peak level, L33, LAeq 1h (dBA)	**Comparator:** Children from noisy classrooms compared to children from noise-abated classrooms and to children from quiet schools. Longitudinal analyses of children who moved from noisy to noise-abated classroom in contrast to children who remained in noisy rooms.	**Confounding**: Number of children in the family, grade in school, months enrolled in school, race, cognitive aptitude test, performance under ambient conditions	**Outcome**:<br>**California Test of Basic Skills – reading and mathematics tests** (California Assessment Program, 1976)<br>**Auditory discrimination test** – ability to discriminate between pairs of words (Wepman, 1958)<br>**Distractibility test –** a crossing out Es test under ambient and distracting conditions (Cohen et al, 1980)<br>**Helplessness** – tested by speed of solving a solvable puzzle after trying to solve an unsolvable puzzle<br>**Perception of noise** – questionnaire for children	**Findings:**<br>Longitudinal testing: children from **noisy schools** were less distractible; more often failed solving the puzzles, solved puzzles for longer time; and had higher levels of noise perception than children in quiet schools.<br>Cross-sectional: children from noisy classrooms more often failed the puzzle test in comparison to children from noise-abated and quiet rooms.<br>**Type of analyses:** Regression analyses<br>**Sample size relating to the effect size:** n=163 for longitudinal analysis, i.e. n=83 from noisy schools and n=80 from quiet schools<br>n=262 for cross-sectional analysis, i.e. n=97 from noisy, n=45 from noise-abated, and n=120 from quiet classrooms	**Comments:**<br>**Findings:**<br>No effect of noise on reading achievement or auditory discrimination between classrooms. No impact of noise on distraction between noisy, abated and quiet classrooms.

5.	Cohen et al, American Scientist, 1981.	See Cohen et l, J Person Social Psychol, 1981						
**Cross-sectional Evidence**								
6.	Haines et al, J Epidemiol Community Health, 2002	**Population**: school children, 123 schools around the airport, total n=11000, aged 11yrs. **Cross-sectional study**	**Noise exposure**: aircraft noise levels **modelled** around schools **Noise source**: aircraft noise **Noise metrics**: LAeq, 16h (dBA) **Noise groups**: schools classified into eight exposure levels depending on aircraft noise contour band (from below 54 to above 72 dBA)	**Comparison**: Children from schools with different noise levels	**Confounding**: Age, gender, social deprivation (free meal), main language spoken at home, child with special need, type of school	**Outcome**: **National Standardized Scores (SATs) for Key Stage 2 in mathematics, science, English** (including four subscales: spelling, handwriting, creative writing and reading)	**Findings:** Increase of **aircraft** noise by contour band was significantly associated with a decrease of the performance in reading test by 0.42 marks (unadjusted), and a decrease in mathematics by 0.73 marks (unadjusted). **Type of analyses:** Multilevel modeling analysis (hierarchical data) **Sample size relating to the effect size:** n=11000	**Comments:** **Findings:** No effect of noise on performance in science and English test subscales. Noise-school performance relationship was influenced by socioeconomic factors.

7.	Green et al, Arch Enviro Health, 1982	**Population**: school children from 362 schools, collected over 5 years (1972-1976), total n=unknown, age unspecified (2nd to 6th grade). **Cross-sectional** study	**Noise exposure**: flight contours **modeled** outside schools **Noise source**: aircraft noise **Noise metrics**: none **Noise groups**: five school groups according to noise exposure contours, each school assigned a noise score	**Comparison**: Dose-response	**Confounding:** Black origin, Puerto Rican origin, absentee rate, eligibility for free lunch, teachers' experience	**Outcomes:** **Reading** – obtained from an annual nationally standardized test; expressed as percentage of students reading 1 or more years below grade level.	**Findings:** **Aircraft** noise exposure was positively correlated with percentage of children reading below grade level. An increase of noise score was associated with an increase of 0.62% in the number of students reading 1 and more years below grade level. **Type of analyses:** Linear regression **Sample size relating to the effect size:** n=unknown	**Comments:** **Findings:** Linear dose-response relationship between noise score values and percent reading below grade level was suggested.
**Road Traffic Noise Exposure**								
**Cross-sectional Evidence**								

8.	Cohen et al, J Experim Soc Psychol, 1973	**Population**: school children, total n=54, 2nd, 3rd, 4th and 5th grade (age not specified);<br>**Sampling procedure**: 73 children approached, exclusion criteria: poor knowledge of English, not living at the building of interest, absence, disturbance on testing day, hearing deficit at audiometric test.<br>**Cross sectional** study	**Noise exposure**: noise levels were **measured** outside the buildings, inside the buildings (hallways), and inside of the apartments (living room)<br>**Noise source**: road traffic<br>**Noise metrics**: not specified (dBA)<br>**Noise groups:** children who lived in the noisy buildings ≥4 years, children who lived in noisy buildings ≤ 3 years	**Comparator**: Children who lived in noisy buildings ≥4 years were compared to children who lived in noisy buildings ≤ 3 years	**Confounding**: Audiometric testing, parent's educational level, floor level, length of residence, school grade, number of children in family	**Outcome**:<br>**Auditory discrimination** – measured by Auditory Discrimination test (Wepman, 1958);<br>**Reading – including word vocabulary, reading comprehension and reading total** tested by the Metropolitan Achievement Reading Test (Durost et al, 1971);<br>**Stroop Color-word test** – Uleman and Reeves, 1971	**Findings:**<br>Children living in **noisy apartments** ≥4 years had positive correlation between auditory discrimination and reading test, positive correlation between floor level and auditory discrimination (adjustment for parent's education); positive correlation between floor and reading test scores (adjustment for parent's education).<br>**Type of analyses:**<br>Correlation, partial correlation, stepwise regression analysis<br>**Sample size relating to the effect size:**<br>n=54 (n=34 living ≥4 years, n=20 living ≤ 3 years in noisy buildings)	**Comments:**<br>**Methods:**<br>Floor level used for noise exposure in analyses but groupings confirmed by noise measurement.<br>**Findings:**<br>Duration of residence is related to impairment in auditory discrimination. Floor level accounts for the variance of auditory discrimination. Auditory discrimination and mother's education account for the total variance in reading test score.<br>**Conclusion:**<br>The role of noise exposure in reading impairment is indirect (through auditory discrimination).

9.	Lukas et al, Report, Department of Health Services, 1981	**Population**: school children, 15 schools socio-economically comparable, total n=1826, age unspecified (3rd and 6th grade).<br>**Cross-sectional** study	**Noise exposure**: noise levels **measured** outside schools, in the community, and indoors (in classrooms)<br>**Noise source**: road traffic noise<br>**Noise metrics**: LAeq 24-hour, L_1, L_{10}, L_{90}, L_{99} (dBA)<br>**Noise groups**: noisy area (L_{99}=53.4 dBA) vs. quiet area (L_{99}=47.5 dBA)	**Comparison**: Quiet vs. noisy schools	**Confounding:** Classroom assignment, socio-economic (income, percentage of poverty), race (White, Black, Hispanic), community noise exposure for each school, English-speaking vs. Non-English-speaking	**Outcomes:**<br>**Reading and mathematics** – tested on Comprehensive Test of Basic Skills (standardized test, School District);<br>**Classroom or grade-level education** – tested on California Assessment Program (California Department of Education)	**Findings:**<br>3rd and 6th-grade pupils from noisy schools had poorer achievement in reading and mathematics in comparison to children from quiet schools. 3rd-graders were 0.4 years behind in reading and 0.2 years behind in mathematics. 6th graders from noisy school were 0.7 years behind in reading.<br>**Type of analyses:** Multiple regression<br>**Sample size relating to the effect size:** n=1826	**Comments:**<br>**Method:**<br>Some quiet schools were exposed to other sources of noise – the selection of school was based on the distance from a freeway, not on actual noise levels.<br>**Findings:**<br>Non-English speaking children had poorer results in reading and mathematics in comparison to English-speaking children.

10.	Shield and Dockrell, J Acoust Soc Am, 2008	**Population**: school children, 142 schools from three boroughs, total n=unspecified, aged 7 and 11yrs.<br>**Cross-sectional study**	**Noise exposure**: ambient noise levels **measured** outside and inside schools<br>**Noise source**: road-traffic noise<br>**Noise metrics**: external noise: LAeq, LAmax, LA90, LA10; internal noise: LAeq, LA90	**Comparison**: Correlation between noise levels and scores in children from suburban and urban schools	**Confounding:** Free school meals, English as an additional language, child with special education needs	**Outcomes:**<br>**National Standardized Scores (SATs) for Key Stage 1** (reading, writing, spelling, mathematics) **and Key Stage 2** (English, science, mathematics)	**Findings:**<br>External noise levels were negatively correlated with all average test scores of KS1 and KS2 in suburban schools, but not in urban schools.<br>**External noise** was significantly associated poor KS1 mathematics in suburban schools, and with poor KS1 and KS2 English in urban schools.<br>**Internal noise** was significantly associated with poor KS1 mathematics, and with all K2 scores.<br>**Type of analyses:** Correlation and regression analysis<br>**Sample size relating to the effect size:** n=unknown	**Comments:**<br>**Findings:**<br>For younger children – correlation was significant with LA90 (i.e. they are affected by external background noise); for older children – correlation was significant with LAmax (more affected by individual external noise events).<br>Negative correlation between noise and scores significant in older children after adjustment (possibly more affected than younger children).

11.	Pujol et al, J Urban Health, 2014	**Population**: school children, 35 schools, different noise exposure at school and home, total n=586, aged 8-9yrs.<br>**Sampling**: 964 children approached, 746 interviewed (response rate 77.4%), 587 with valid tests<br>**Exclusion criteria**: change of residence, hearing impairment<br>**Cross sectional** study	**Noise exposure**: noise levels **modelled** outside homes (24 hours) and schools (from 6 am to 6 pm)<br>**Noise source**: ambient noise, predominantly road traffic<br>**Noise metrics**: At school: LAeq, day (dBA)<br>At home: Lden (dBA)	**Comparator**: lower levels of noise at home and at school	**Confounding:** Household socio-economic status, employment, parental educational level, age, sex, main language spoken at home, reading as leisure activity	**Outcome:** **National standardized assessment test in French and mathematics**	**Findings:**<br>**Ambient noise** levels **at home** were negatively associated with scores in French (unadjusted), but not with mathematics score.<br>**Ambient noise** levels **at school** were negatively associated with scores in French (unadjusted and adjusted), and with mathematics score (unadjusted for confounders).<br>Increase of noise by 1 dBA **at school** was associated with a decrease of French score and mathematics score by approx 0.5 points.<br>**Type of analyses:** Multilevel linear regression model<br>**Sample size relating to the effect size:** n=587 with complete data in French, n=586 with complete data in mathematics	**Comments:**<br>**Findings:** Children who already repeated a school year were exposed to higher Lden at homes than the other pupils (by 2 dBA)
**Railway Noise Exposure**								
**Intervention Evidence**								

12.	Bronzaft, J Enviro Psychol, 1981	**Population**: school children, first testing: n=350, 2nd, 3rd, 5th and 6th grade (age not specified); second testing: n=605, 2nd, 3rd, 5th and 6th grade (age not specified); noisy and quiet classes matched for intelligence and achievement and teaching method. **Cross-sectional study:** comparison between noisy, and quiet classrooms before (in 1978) and after noise abatement (in 1980/1981) **Intervention:** noise abatement measures performed at the source (change or rubber pads for the tracks) and inside school (sound absorbing ceilings installed)	**Noise exposure**: noise levels were **measured** inside one classroom of the school **Noise source**: subway trains running at elevated tracks **Noise metrics**: not specified (dBA) **Noise groups:** noisy classrooms (during train passing L=89 dBA) and quiet classrooms (noise level not specified). After noise abatement intervention noise levels reduced in noisy classrooms by 6-8 dBA (during train passing L=81-83 dBA)	**Comparator:** Children from noisy classrooms compared to children from the quiet classrooms	**Confounding**: none	**Outcome**: **Reading achievement –** tested by the California Achievement Test (CTB-McGraw hill, 1977)	**Findings:** **Before noise abatement:** Children on the noisy side of the school had poorer reading achievement compared to children from quiet classrooms. **After noise abatement:** Children on both sides of the building had similar reading scores. **Type of analyses:** Analysis of variance; Chi-square test **Sample size relating to the effect size:** n=350 at first testing, n=605 at second testing	**Comments:** **Methods:** This is not a follow-up study because children change classrooms every year – the two samples (before and after abatement) were independent
**Cross-sectional Evidence**								

13.	Bronzaft and McCarthy, Enviro Behav, 1975	**Population**: school children, total n=161, 2nd, 4th and 6th grade (age not specified); children from noisy and quiet classes (total n=14 classes) matched for intelligence and achievement test. **Cross sectional** study	**Noise exposure**: noise levels were **measured** inside one classroom of the school **Noise source**: subway trains running at elevated tracks **Noise metrics**: not specified (dBA) **Noise groups:** noisy classrooms (average L=59 dBA; during train passing L=89 dBA) and quiet classrooms (noise level not specified)	**Comparator**: Children and classes on the noisy side of school compared to children and classes located on the quiet side of school	**Confounding**: none	**Outcome**: **Attitudes toward the noise** designed for the purposes of the study (similar to Fitzroy and Reid, 1963) available for 212 children **Reading achievement – including word knowledge, reading comprehension and general reading** tested by the Metropolitan Achievement Reading Test (Durost et al, 1971) – available for 161 children and average values available for all classes	**Findings: Children** on the noisy side of the school had poorer reading achievement performance (word knowledge and reading comprehension) compared to children from quiet classrooms. **Classes** on the noisy side of the building had lower mean reading scores than classes on quiet side of the school. **Type of analyses:** Analysis of variance; Chi-square test **Sample size relating to the effect size:** n=212 children tested individually; n=14 classes of children with average scores	**Comments: Findings:** Children on noisy side felt that there was too much noise in the classroom, that noise made it hard for them to do their work, and that subway trains bothered them or made it hard for them to think. They also more often rated their classroom as noisy or very noisy.

Table S3: Characteristics of included studies for environmental §noise effects on long-term & short-term memory

	Reference	**Population**: general population in settings (hospitals, residences, public venues, educational facilities) + **response rate** and other **selection** /bias factors **Cross-sectional or longitudinal**	**Exposure**: exposure to high levels of environmental noise from various noise sources + noise metric involved + modelled or measured noise	**Comparator**: no noise exposure or lower levels of noise exposure	**Confounding**: adjusted for confounding	**Outcome**: assessment of outcome	**Findings:** expressed as effect per dB if possible.<br><br>Type of analyses<br><br>Sample size relating to the effect size	**Comments:** Anything else to note
**Aircraft Noise Exposure**								
**Intervention Evidence**								

1.	Hygge et al, Psychol Sci, 2002	**Population**: school children, noisy and quiet schools around the old and the new airport, total n=326, aged 8-12yrs. Four groups: n=43 old-airport, no-noise; n=65 old-airport plus noise; n=107 new-airport, no-noise; n=111 new-airport plus noise **Inclusion criteria**: 2 years of residence, speaking German fluently, normal hearing (assessed by audiometric screening) **Longitudinal study;** baseline (wave 1): 6 months before the opening of new airport, follow-up 1 and 2 years (waves 2 and 3) after changeover of airports **Intervention Study**	**Noise exposure**: aircraft noise levels **measured** around schools before and after relocation of the airport; **Noise source**: aircraft noise **Noise metrics**: Leq, 24h (dBA)	**Comparison**: children from noisy schools around the old and the new airport compared to children from quiet schools around the old and the new airport	**Confounding**: None	**Outcomes: Reading test** in German (Biglmaier, 1969), **Long-term memory** – recall of a text read in noisy conditions the previous day, **Short-term memory** – remembering consonants), **Attention** – visual search and reaction time were tested in both quiet and noisy conditions, **Speech perception** – hearing a story against noisy background (adapted from Hygge, Rönnberg, Larsby, Arlinger, 1992)	**Findings: At the new airport** children showed a decrease of the number of correct answers on long-term memory task, and impairment of speech perception. **At the old airport** children showed decrease in the number of errors on reading test, increase of the number of correct answers on short-term memory task, and improvement of speech perception. **Type of analyses:** t-test for independent samples, multivariate analyses of variance MANOVA **Sample size relating to the effect size:** n=326	**Comments: Findings:** The effect of noise on the reading tasks was not mediated by memory or speech perception. Poorer reading was not mediated by speech perception, and impaired recall was in part mediated by reading.

**Longitudinal evidence**								
	See Hygge et al, Psychol Sci, 2002 above							
**Cross-sectional evidence**								

2.	Clark et al, Am J Epidemol, 2012	**Population**: school children, total n=960, aged 9-10yrs. **Cross sectional** study	**Noise exposure**: aircraft noise levels at home and at school **measured** in an area from 7 am to 11 pm; road traffic noise at school **combined** from measurements and models **Noise source**: aircraft and road traffic **Noise metrics**: LAeq, 16h (dBA)	**Comparator**: lower levels of noise exposure	**Confounding**: Modelled concentrations of **NO2** (µg/m3); Socio-economic status (employment, housing tenure, home crowding), maternal education, ethnicity, main language spoken at home; age, gender, long-standing illness, parental support for schoolwork, classroom glazing; other noise exposure source	**Outcome**: **Reading comprehension** Suffolk Reading Scale Level 2 (SRS2) (Hagley, 2002), CITO Readibility Index for elementary and Special Education (Staphorsius, 1994), Ecaluacion Comprension Lectora ECL-2 (De La Cruz, 1999); **Episodic memory** tested with Child Memory scale, (Cohen, 1997)	**Findings:** Increase of **aircraft** noise at school by 1 dBA correlated to decrease of the performance on the following tests: reading comprehension (by 0.01 marks), recognition memory (by 0.045 marks), information recall (by 0.043 marks) and conceptual recall (by 0.015 marks). **Type of analyses:** Multilevel linear and logistic regression models **Sample size relating to the effect size:** n=719 with air pollution data and n=241 without air pollution data	**Comments:** **Findings:** Moderate correlation between NO2 levels and aircraft noise and road traffic noise. No association between road traffic noise and reading comprehension

3.	Haines, Stansfeld, Brentnall et al, Psychol Med, 2001	**Population**: school children, 20 schools, total n=451, aged 8-11yrs. Noisy and quiet schools matched for age, sex, other noise sources, noise protection at school, socio-economic status, main language; Response rate 82% **Cross-sectional study**	**Noise exposure**: aircraft noise levels **modelled** around schools **Noise source**: aircraft noise **Noise metrics**: LAeq, 16h (dBA) **Noise groups**: noisy schools (Leq, 16h >63 dBA), quiet schools (Leq, 16h <57 dBA)	**Comparator**: Children from high aircraft noise schools compared with children from lower aircraft noise level schools	**Confounding**: Household deprivation score – incorporating income, home tenure, car ownership, employment, central heating, social class and household crowding (Townsend, et al, 1989), age, main language spoken at home	**Outcome**: **Reading comprehension** – Suffolk Reading Scale (SRS2) Level 2 (Hagley, 2002); **Long-term memory** – including immediate recall, delayed recall, and recognition – adapted from Child Memory Scale (Cohen, 1997); **Backward serial digit recall** (Pickering and Gathercole, 2000); **Sustained Attention** – score task from Tests of Everyday Attention for Children (TEA-Ch) (Manly et al, 1998)	**Findings:** Children in high noise schools had poor performance on the difficult items (20% of all items) of the reading scale. **Type of analyses:** Analysis of covariance (ANCOVA), Multilevel modeling analysis **Sample size relating to the effect size:** n=451 (n=236 from high-level noise schools, n=215 from low-level noise schools)	**Comments:** **Findings:** No difference in reading, immediate recall, delayed recall, recognition memory, sustained attention and serial backward digit recall between children from high-level aircraft noise schools and children from low level noise schools.

4.	Haines, Stansfeld, Job et al, Psychol Med, 2001	**Population**: school children, 8 schools, total n=340, aged 8-11yrs. Noisy and quiet schools matched for age, sex, other noise sources, noise protection at school, socio-economic status, ethnic groups; Response rate 77%<br>**Cross-sectional study**	**Noise exposure**: aircraft noise levels **modelled** around schools; acute noise levels **measured** indoors<br>**Noise source**: aircraft noise<br>**Noise metrics**: LAeq, 16h (dBA) outdoors; single event noise exposure level (SEL dBA); LAeq during time needed for task completion<br>**Noise groups**: noisy schools (Leq, 16h >66 dBA), quiet schools (Leq, 16h <57 dBA)	**Comparator**: Children from high aircraft noise schools compared with children from lower aircraft noise level schools	**Confounding**: Household deprivation score – incorporating income, crowding, home ownership, unemployment, (Townsend, et al, 1989), age, main language spoken at home	**Outcome**:<br>**Reading comprehension** – Suffolk Reading Scale (SRS2) Level 2 (Hagley, 2002);<br>**Long-term memory** – including recognition task and recall task (Evans et al, 1995);<br>**Short-term memory** – serial digit recall task;<br>**Motivation – performance measure**, i.e. solving insolvable and solvable puzzle (Evans et al, 1995);<br>**Child Attributional Style** – measured by Child Attribution Style Questionnaire (CASQ; Kaslow & Nolen-Hoeksema, 1991);<br>**Classroom motivation, i.e. learned helplessness** – estimated by teachers using Student Behaviour Checklist (SBC; Fincham et al. 1989)	**Findings:**<br>Children in high noise schools had poor performance on the reading scale than children from low noise schools (even after adjustment for deprivation, age, language).<br>Children in high noise schools had poor long-term memory than children from low noise schools (only a subsample without bias).<br>No effect of noise exposure on motivation, self-reported attributional scale and classroom motivation.<br>**Type of analyses:** Analysis of covariance (ANCOVA)<br>**Sample size relating to the effect size:** n=340 (n=169 from high-level noise schools, n=171 from low-level noise schools)	**Comments:**<br>**Findings:**<br>Acute aircraft noise exposure at the time of testing was not associated with reading comprehension or annoyance.<br>**Conclusion:**<br>The association between aircraft noise exposure and reading comprehension is independent from noise annoyance, acute noise levels, age, language, and deprivation.

5.	Stansfeld et al, Lancet, 2005	**Population**: school children, 89 schools around three airports, total n=2844, aged 9-13yrs. **Cross sectional** study	**Noise exposure**: aircraft noise levels at home and at school **measured** in an area from 7 am to 11 pm; road traffic noise at school **modelled** or **combination** of measurements with models **Noise source**: aircraft and road traffic **Noise metrics**: LAeq, 16h (dBA)	**Comparator**: lower levels of noise exposure	**Confounding**: age, gender, parental employment, home ownership, home crowding, mother's educational attainment, long-standing illness, main language spoken at home, parental support for school work, classroom glazing	**Outcome**: **Reading comprehension** Suffolk Reading Scale Level 2 (SRS2) (Hagley, 2002), CITO Readibility Index for elementary and Special Education (Staphorsius, 1994), Ecaluacion Comprension Lectora ECL-2 (De La Cruz, 1999); **Episodic memory** – Child Memory scale, (Cohen, 1997); **Sustained attention** – Toulouse Pieron test (Toulouse et al, 1986); **Working memory** – Search and Memory test (Smith and Miles, 1987; Hygge et al, 2003); **Prospective memory** (writing initials as instructed)	**Findings:** Increase of **aircraft** noise at school by 1 dBA correlated to decrease of the performance on the reading test by 0.008 marks; Increase of aircraft noise at school by 1 dBA correlated to decrease of the performance on the recognition test by 0.018 marks. Increase of **road traffic** noise by 1 dB was associated with an increase of information recall by 0.038 marks and an increase of conceptual recall by 0.013 marks. **Type of analyses:** Multilevel model analyses (for data clustering) **Sample size relating to the effect size:** range from 1939 to 2014 with complete data	**Comments:** **Findings:** No association between aircraft and road traffic noise and cued conceptual recall, cued information recall, prospective memory, working memory, and sustained attention.

6.	Stansfeld, Hygge, Clark et al, Noise Health, 2010	**Population**: school children, two studies re-analysed.<br>**Munich study**: n=326, aged 9-11yrs, around old and new airport<br>Inclusion criteria: 2 years of residence, speaking German fluently<br>**Longitudinal study;** baseline (wave 1): 6 months before the opening of new airport, follow-up 1 and 2 years (waves 2 and 3) after changeover of airports<br>**RANCH study:** n=857, aged 9-10yrs, around the airport<br>**Cross-sectional** study	**Noise exposure**: aircraft noise levels **measured** around schools in Munich study; **modeled** in RANCH study<br>**Noise source**: aircraft noise<br>**Noise metrics**: Munich study: Leq, 24h (dBA);<br>RANCH study: Leq, nighttime at home (dBA) (11 p.m. to 7 a.m.)	**Comparison**: Munich study: children from noisy schools around the old and the new airport compared to children from quiet schools around the old and the new airport.<br>RANCH study: higher levels of aircraft noise exposure compared to lower levels of noise	**Confounding**: Munich study: self-reported sleep quality; RANCH study: daytime aircraft noise at school, road-traffic noise, sleep problems, age, sex, parental employment, crowding, homeownership, mother's education, child's illness, main language at home, parental support for home work, classroom glazing	**Outcomes:**<br>**Munich study:** **Reading test** in German (Biglmaier, 1969) in noisy conditions (80 dBA)<br>**Long-term memory** – recall of a text read in noisy conditions the previous day.<br>**RANCH study:** **Reading comprehension** Suffolk Reading Scale Level 2 (SRS2) (Hagley, 2002), **Episodic memory** – Child Memory Scale, (Cohen, 1997); **Sustained attention** – Toulouse Pieron test (Toulouse et al, 1986);<br>**Working memory** – Search and Memory test (Smith and Miles, 1987; Hygge et al, 2003)	**Findings:**<br>**Munich study:** No association between **nighttime aircraft** noise and cognitive impairment (mediated by sleep quality).<br>**RANCH study:** Increase of **nighttime aircraft** noise at home by 1 dBA correlated to decrease of the reading test performance by 0.009 marks, and to decrease of recognition memory by 0.031 marks.<br>**Type of analyses:** Multilevel modeling<br>**Sample size relating to the effect size:** n=326 (Munich study), n=842 for RANCH study	**Comments:**<br>**Findings:**<br>RANCH study: Daytime and nighttime noise exposure highly correlated.<br>Nighttime aircraft exposure was no longer associated with impaired reading comprehension and recognition memory after adjustment for daytime aircraft noise at school.

7.	Matheson et al, Noise Health, 2010	**Population**: school children, 89 schools around three airports, total n=2844, aged 9-13yrs.<br>**Sampling**: 3207 children approached; 2844 pupils participated; response rate: 89% of children, 80% of parents<br>**Cross sectional** study	**Noise exposure**: aircraft noise levels at home and at school **measured** or **modeled** in an area; road traffic noise at school **combined** from measurements and models; acute noise measurements<br>**Noise source**: aircraft and road traffic<br>**Noise metrics**: LAeq, 16h (dBA)	**Comparator**: lower levels of noise exposure	**Confounding**: age, gender, country, socio-economic status (employment, home ownership, home crowding), mother's educational attainment, long-standing illness, main language spoken at home, parental support for school work, other noise source; dyslexia, acute noise during testing	**Outcome**:<br>**Episodic memory** (including information recall, conceptual recall, recognition memory) tested with Children's Memory scale (Cohen, 1997);<br>**Prospective memory** test (Shield and Dockrell, 2002)	**Findings:**<br>Increase of **road traffic** noise by 1 dB was associated with an increase of information recall by 0.038 marks and an increase of conceptual recall by 0.013 marks.<br>Increase of **aircraft** noise by 1 dB was associated with a decrease of recognition memory by 0.018 marks but was not related to prospective memory.<br>**Type of analyses:** Multilevel linear regression analyses<br>**Sample size relating to the effect size:** n=2844 with complete data	**Comments:**<br>**Findings:**<br>Aircraft noise was not significantly related to either information recall or conceptual recall. Road traffic noise was not significantly related to either recognition memory or prospective memory.

8.	Matsui et al, Noise Health, 2004	**Population**: school children, high aircraft noise exposure at school, three groups of aircraft noise exposure at home; total n=451, aged 8-9yrs, response rate: 83% in high noise schools and 81% in low noise schools **Cross sectional** study	**Noise exposure**: aircraft noise levels at home and at school **modelled** **Noise source**: aircraft noise **Noise metrics**: LAeq, 16h (dB) **Noise groups**: All children attending schools with high levels of aircraft noise (LAeq, 16h > 63 dB), but with different levels of aircraft noise at home (LAeq, 16h < 63dB vs. LAeq, 16h =63-66 dB, vs. LAeq, 16h > 63 dB)	**Comparator**: children from homes with low noise levels (LAeq, 16h < 63 dB) compared to children from schools with higher noise levels (LAeq, 16h =63-66 dB), and to the highest noise levels (LAeq, 16h > 63 dB)	**Confounding:** Age, sex, schools, household deprivation score (including income, home tenure, car ownership, employment, central heating, household crowding, social class), mother's educational level, long-standing illness, main language spoken at home	**Outcome**: **Reading comprehension** Suffolk Reading Scale Level 2 (Hagley, 1987); **Long term Memory Recall and Recognition** – tested with Children's Memory scale (Cohen, 1997); **Sustained Attention** tested with Tests of Everyday Attention for Children (TEA-Ch) (Manly et al, 1998)	**Findings:** **Aircraft** noise levels at home significantly predict poor performance at immediate recall and delayed recall tests. **Type of analyses:** Multiple logistic regression analyses **Sample size relating to the effect size:** n=163 with complete data	**Findings:** No association between aircraft noise levels at home and delayed recognition, sustained attention and reading comprehension.

9.	van Kempen et al, Enviro Health, 2010	**Population**: school children from 24 schools around Amsterdam airport, 620 children approached, total sample n=553, aged 9-11yrs<br>**Cross-sectional** study	**Noise exposure**: home and school addresses for each children linked to **modelled** noise levels<br>**Noise source**: aircraft and road traffic<br>**Noise metrics:** LAeq 7-23h (dBA) for aircraft noise; LAeq 7-23h (dBA) for road traffic noise	**Compared to:** lower levels of noise exposure	**Confounding**: Age, gender, main language spoken at home, long-standing illness, parental support for school work, school glazing, socio-economic status (crowding, home ownership, parental employment, mother's education), the other noise source	**Outcome**:<br>1) **Neurobehavioral evaluation system (NES) tests** (Letz, 1001): Simple Reaction Time Test, Switching Attention Test, Hand-Eye-Coordination Test, Symbol-Digit Substitution Test, Digit Memory Span Test.<br>2) **Other cognitive outcomes**: Reading comprehension (CRIE test, Staphorsius, 1994); Prospective memory (writing initials where instructed); Episodic memory (Child Memory scale, Cohen, 1997); Working memory (Search and Memory test, Hygge et al, 2003); Sustained attention (Toulouse Pieron test, Toulouse et al, 1986)	**Findings:**<br>Positive and significant relation between **aircraft** noise exposure at school and the number of errors on Switching Attention test (SAT).<br>Positive and significant relation between **road traffic** noise at school and the number of errors on SAT test<br>**Type of analyses**: multilevel model analyses by MIXED procedure<br>**Sample size relating to the effect size:** n=433 children with complete data	**Comments:**<br>**Findings:**<br>Significant correlation between aircraft and road traffic noise levels.<br>No relation between aircraft noise exposure at school and other NES tests; no relation between aircraft noise exposure at home and cognitive outcomes.<br>No relation between road traffic noise exposure at home and cognitive outcomes.

10.	van Kempen et al, Enviro Res, 2012	**Population**: school children from 24 schools around Amsterdam airport, 620 children approached, total sample n=553, aged 9-11yrs<br>**Cross-sectional** study	**Noise exposure**: home and school addresses for each children linked to **modelled** noise levels<br>**Noise source**: aircraft and road traffic<br>**Noise metrics:** LAeq 7-23h (dBA) for aircraft noise; LAeq 7-23h (dBA) for road traffic noise	**Compared to:** lower levels of noise exposure	**Confounding**: Modelled concentrations of **NO2** (µg/m3) and particulate matter (**PM10**);<br>Age, gender, main language spoken at home, long-standing illness, parental support for school work, school glazing, socio-economic status (crowding, home ownership, parental employment, mother's education)	**Outcomes:**<br>**Neurobehavioral evaluation system (NES) tests** (Letz, 1001): Simple Reaction Time Test, Switching Attention Test, Hand-Eye-Coordination Test, Symbol-Digit Substitution Test, Digit Memory Span Test.	**Findings:**<br>Positive and significant relation between **road traffic** noise at school and the number of errors on SAT test; Positive and significant relation between **aircraft** noise at school and the number of errors on SAT test; both significant after adjustment for NO2 concentration at school.<br>**Type of analyses**: multilevel model analyses by MIXED procedure<br>**Sample size relating to the effect size:** n=485 children with complete data	**Comments:**<br>**Findings:**<br>Positive correlation between aircraft and road traffic noise levels at home and school; positive correlation between NO2 and PM10 at home and school. NO2 level at school significantly associated with span length measured with DMST.<br>No relation between aircraft and road traffic noise exposure at home and cognitive outcomes.<br>Combined exposure to NO2 and road traffic noise had effect on reaction times measured with SRTT and the number of errors on SAT.

11.	Klatte et al, Report, NORAH study, 2014	**Population**: 85 second-grade classes from 29 primary schools around Frankfurt/Main airport; children: total n=1243, age 8yrs, 4months<br>**Cross-sectional** study	**Noise exposure:** Noise contours at school and at home around airport<br>**Noise source:** aircraft noise<br>**Noise metrics:** Leq around schools (time 8-14h); average 49.5 dB, Median 50.6 dB; range 39 to 59 dB; Leq at home (6-18h)<br>**Noise groups:**<br>3 groups at school: low exposure < 47 dB; middle exposure 47 to < 55 dB; high exposure ≥ 55 dB	**Comparison:** Change estimate per 10 dBA increase	**Confounding:** Migrant background. Children´s exposure to road traffic and railway noise. Also used as confounders: auditory thinking, phonological awareness, episodic memory. Class characteristics (size, proportion of migrants, socioeconomic status, parental engagement), classroom reverberation and insulation.	**Outcomes:**<br>**Reading test**: standardized comprehension test for primary school children (ELFE 1-6, Lenhard & Schneider, 2006).<br>**Language functions** – speech perception, auditory thinking, phonological short-term memory, phonological awareness, episodic memory test.<br>**Other outcomes**: Health-related quality of life, well-being at home and school, noise annoyance at home and school.	**Findings:**<br>A 10 dB increase in aircraft noise associated with a decrement of one-tenth of an SD on the **reading** test, corresponding to a one month reading delay in this test.<br>Similar results for subscales: word and text comprehension.<br>Similar results found for aircraft noise at home.<br>No association between aircraft noise exposure (39-59dB LAeq 8am-2pm) and a test of phonological short-term memory<br>**Type of analyses:** Multilevel regression<br>**Sample size relating to the effect size:**<br>n=1090	**Comments:**<br>**Methods:**<br>Impossible to separate home and schools exposure (high correlation).<br>**Findings:**<br>Significant confounding of migrant background – aircraft noise (both home and school) strongly affects **reading** comprehension of children without migration background (1.5-2.5 months reading delay).<br>No effect of aircraft noise on tested **language** functions.<br>Aircraft noise at school and at home significantly associated with **annoyance**.<br>Aircraft noise was associated with less positive judgments of health-related **quality of life**, well-being at school, and sleep quality.

**ROAD TRAFFIC NOISE EXPOSURE**								
**Cross-sectional Evidence**								
	See Stansfeld et al, Lancet, 2005 above							
	See Stansfeld, Hygge, Clark et al, Noise Health, 2010 above							
	See Matheson et al, Noise Health, 2010 above							
	See van Kempen et al, Enviro Health, 2010 above							
	See van Kempen et al, Enviro Res, 2012 above							
**RAILWAY NOISE EXPOSURE**								
**Cross-sectional Evidence**								

12.	Lercher et al, Enviro and Behav, 2003	**Population**: school children, total n=123, aged 9-10yrs. **Selection**: two neighborhoods selected from larger sample based on noise levels **Cross-sectional** study	**Noise exposure**: not specified whether noise levels were measured or modeled **Noise source**: rail and road traffic noise **Noise metrics**: Ldn **Noise groups**: noisy neighborhood (Ldn=62 dBA), and quiet neighborhood (Ldn=46.1 dBA)	**Comparison:** Children exposed to higher noise levels compared to children exposed to lower levels	**Confounding:** none	**Outcomes:** **Attention** – visual search task; **Intentional, explicit memory** – reading a story (Seyfried, 1998); **Incidental memory** – free recall; **Motivation** – solving insolvable and solvable puzzle (Evans et al, 2001)	**Findings:** No effect of noise on visual search performance (attention). Children from high noise group had poorer results on explicit and incidental memory tests. **Type of analyses:** Chi-square, t-test **Sample size relating to the effect size:** n=123	**Comments:** **Findings:** No adjustment for age, gender, parent's education, housing characteristics etc reported in the paper.

Table S4: Characteristics of included studies for environmental noise effects on attention

	Reference	**Population**: general population in settings (hospitals, residences, public venues, educational facilities) + **response rate** and other **selection** /bias factors **Cross-sectional or longitudinal**	**Exposure**: exposure to high levels of environmental noise from various noise sources + noise metric involved + modelled or measured noise	**Comparator**: no noise exposure or lower levels of noise exposure	**Confounding**: adjusted for confounding	**Outcome**: assessment of outcome	**Findings:** expressed as effect per dB if possible.<br><br>Type of analyses<br><br>Sample size relating to the effect size	**Comments:** Anything else to note
**AIRCRAFT NOISE EXPOSURE**								
**INTERVENTION EVIDENCE**								
1.	Hygge et al, Psychol Sci, 2002	**Population**: school children, noisy and quiet schools around the old and the new airport, total n=326, aged 8-12yrs. Four groups: n=43 old-airport, no-noise; n=65 old-airport plus noise; n=107 new-airport, no-noise; n=111 new-airport plus noise	**Noise exposure**: aircraft noise levels **measured** around schools before and after relocation of the airport; **Noise source**: aircraft noise **Noise metrics**: Leq, 24h (dBA)	**Comparison**: children from noisy schools around the old and the new airport compared to children from quiet schools around the old and the new airport	**Confounding**: None	**Outcomes: Reading test** in German (Biglmaier, 1969), **Long-term memory** – recall of a text read in noisy conditions the previous day, **Short-term memory** – remembering consonants), **Attention** – visual search and reaction time were	**Findings: At the new airport** children showed a decrease of the number of correct answers on long-term memory task, and impairment of speech perception. **At the old airport** children showed decrease in the number of errors on reading test,	**Comments: Findings:** The effect of noise on the reading tasks was not mediated by memory or speech perception. Poorer reading was not mediated by speech perception, and impaired recall was in part mediated by

		**Inclusion criteria**: 2 years of residence, speaking German fluently, normal hearing (assessed by audiometric screening) **Longitudinal study;** baseline (wave 1): 6 months before the opening of new airport, follow-up 1 and 2 years (waves 2 and 3) after changeover of airports **Intervention Study**				tested in both quiet and noisy conditions, **Speech perception** – hearing a story against noisy background (adapted from Hygge, Rönnberg, Larsby, Arlinger, 1992)	increase of the number of correct answers on short-term memory task, and improvement of speech perception. **Type of analyses:** t-test for independent samples, multivariate analyses of variance MANOVA **Sample size relating to the effect size:** n=326	reading.
**Longitudinal evidence**								
2.	Haines et al, Int J Epidemiol, 2001	**Population**: school children, noisy and quiet schools around the airport, total n=275, aged 8-11yrs. **Sampling procedure**: baseline sample tested in 1996: n=340 children;	**Noise exposure**: aircraft noise levels **modelled** around schools; acute noise exposure measured inside classrooms **Noise source**: aircraft noise **Noise metrics**: LAeq, 16h (dBA)	**Comparison**: children from noisy schools around compared to children from quiet schools	**Confounding**: Age, household deprivation score – incorporating income, home ownership, unemployment, adapted from Townsend's Scale (Townsend, et al, 1989), main language spoken	**Outcome**: **Reading comprehension** – Suffolk Reading Scale (SRS2) Level 2 and Level 3 (Hagley, 2002) **Sustained Attention** – score task from Tests of Everyday Attention for Children (TEA-	**Findings:** Children from high-**aircraft** noise schools had poorer reading comprehension and poorer sustained attention. **Type of analyses:** Analyses of covariance	**Comments:** At follow-up, reading comprehension was poorer among children from high-aircraft noise schools, but non-significant after adjustment. This implies that

		follow-up sample tested in 1997: response rate 81%, n=275 **Longitudinal study** at two time points	**Noise groups**: High-aircraft noise-impact schools (Leq, 16h >66 dBA), low-aircraft noise-impact schools (Leq, 16h <57 dBA)		at home	Ch) (Manly et al, 1998)	ANCOVA **Sample size relating to the effect size:** n=148 from high-aircraft noise exposed schools, n=127 from low-aircraft noise exposed schools	the effect of noise on reading comprehension may be influenced by socio-demographic factors.
	See Hygge et al, Psychol Sci, 2002 above							
**Cross-sectional evidence**								
3.	Haines, Stansfeld, Brentnall et al, Psychol Med, 2001	**Population**: school children, 20 schools, total n=451, aged 8-11yrs. Noisy and quiet schools matched for age, sex, other noise sources, noise protection at school, socio-economic status, main language; Response rate 82% **Cross-sectional study**	**Noise exposure**: aircraft noise levels **modelled** around schools **Noise source**: aircraft noise **Noise metrics**: LAeq, 16h (dBA) **Noise groups**: noisy schools (Leq, 16h >63 dBA), quiet schools (Leq, 16h <57 dBA)	**Comparator**: Children from high aircraft noise schools compared with children from lower aircraft noise level schools	**Confounding**: Household deprivation score – incorporating income, home tenure, car ownership, employment, central heating, social class and household crowding (Townsend, et al, 1989), age, main language spoken at home	**Outcome**: **Reading comprehension** – Suffolk Reading Scale (SRS2) Level 2 (Hagley, 2002); **Long-term memory** – including immediate recall, delayed recall, and recognition – adapted from Child Memory Scale (Cohen, 1997); **Backward serial digit recall** (Pickering and Gathercole, 2000); **Sustained Attention** – score	**Findings:** Children in high noise schools had poor performance on the difficult items (20% of all items) of the reading scale. **Type of analyses:** Analysis of covariance (ANCOVA), Multilevel modeling analysis **Sample size relating to the effect size:** n=451 (n=236 from high-level noise schools, n=215 from low-level	**Comments:** **Findings:** No difference in reading, immediate recall, delayed recall, recognition memory, sustained attention and serial backward digit recall between children from high-level aircraft noise schools and children from low level noise schools.

						task from Tests of Everyday Attention for Children (TEA-Ch) (Manly et al, 1998)	noise schools)	
4.	Stansfeld et al, Lancet, 2005	**Population**: school children, 89 schools around three airports, total n=2844, aged 9-13yrs. **Cross sectional** study	**Noise exposure**: aircraft noise levels at home and at school **measured** in an area from 7 am to 11 pm; road traffic noise at school **modelled** or **combination** of measurements with models **Noise source**: aircraft and road traffic **Noise metrics**: LAeq, 16h (dBA)	**Comparator**: lower levels of noise exposure	**Confounding**: age, gender, parental employment, home ownership, home crowding, mother's educational attainment, long-standing illness, main language spoken at home, parental support for school work, classroom glazing	**Outcome**: **Reading comprehension** Suffolk Reading Scale Level 2 (SRS2) (Hagley, 2002), CITO Readibility Index for elementary and Special Education (Staphorsius, 1994), Ecaluacion Comprension Lectora ECL-2 (De La Cruz, 1999); **Episodic memory** – Child Memory scale, (Cohen, 1997); **Sustained attention** – Toulouse Pieron test (Toulouse et al, 1986); **Working memory** – Search and Memory test (Smith and Miles, 1987; Hygge et al, 2003); **Prospective memory** (writing	**Findings:** Increase of **aircraft** noise at school by 1 dBA correlated to decrease of the performance on the reading test by 0.008 marks; Increase of aircraft noise at school by 1 dBA correlated to decrease of the performance on the recognition test by 0.018 marks. Increase of **road traffic** noise by 1 dB was associated with an increase of information recall by 0.038 marks and an increase of conceptual recall by 0.013 marks. **Type of analyses:**	**Comments:** **Findings:** No association between aircraft and road traffic noise and cued conceptual recall, cued information recall, prospective memory, working memory, and sustained attention.

						initials as instructed)	Multilevel model analyses (for data clustering)<br>**Sample size relating to the effect size:** range from 1939 to 2014 with complete data	
5.	Stansfeld, Hygge, Clark et al, Noise Health, 2010	**Population**: school children, two studies re-analysed.<br>**Munich study**: n=326, aged 9-11yrs, around old and new airport<br>Inclusion criteria: 2 years of residence, speaking German fluently<br>**Longitudinal study;** baseline (wave 1): 6 months before the opening of new airport, follow-up 1 and 2 years (waves 2 and 3) after changeover of airports<br>**RANCH study:** n=857, aged 9-	**Noise exposure**: aircraft noise levels **measured** around schools in Munich study; **modeled** in RANCH study<br>**Noise source**: aircraft noise<br>**Noise metrics**: Munich study: Leq, 24h (dBA);<br>RANCH study: Leq, nighttime at home (dBA) (11 p.m. to 7 a.m.)	**Comparison**: Munich study: children from noisy schools around the old and the new airport compared to children from quiet schools around the old and the new airport.<br>RANCH study: higher levels of aircraft noise exposure compared to lower levels of noise	**Confounding**: Munich study: self-reported sleep quality; RANCH study: daytime aircraft noise at school, road-traffic noise, sleep problems, age, sex, parental employment, crowding, homeownership, mother's education, child's illness, main language at home, parental support for home work, classroom glazing	**Outcomes:**<br>**Munich study:** **Reading test** in German (Biglmaier, 1969) in noisy conditions (80 dBA)<br>**Long-term memory** – recall of a text read in noisy conditions the previous day.<br>**RANCH study:** **Reading comprehension** Suffolk Reading Scale Level 2 (SRS2) (Hagley, 2002),<br>**Episodic memory** – Child Memory Scale, (Cohen, 1997); **Sustained attention** – Toulouse Pieron test (Toulouse et	**Findings:**<br>**Munich study:** No association between **nighttime aircraft** noise and cognitive impairment (mediated by sleep quality).<br>**RANCH study:** Increase of **nighttime aircraft** noise at home by 1 dBA correlated to decrease of the reading test performance by 0.009 marks, and to decrease of recognition memory by 0.031 marks.<br>**Type of analyses:** Multilevel modeling	**Comments:**<br>**Findings:**<br>RANCH study: Daytime and nighttime noise exposure highly correlated.<br>Nighttime aircraft exposure was no longer associated with impaired reading comprehension and recognition memory after adjustment for daytime aircraft noise at school.

		10yrs, around the airport **Cross-sectional** study				al, 1986); **Working memory** – Search and Memory test (Smith and Miles, 1987; Hygge et al, 2003)	**Sample size relating to the effect size:** n=326 (Munich study), n=842 for RANCH study	
6.	Cohen et l, J Person Social Psychol, 1981<br><br>Cohen et al, American Scientist, 1981.	**Population**: school children, noisy and quiet schools around the airport, first testing n=262, second testing n=163, age unknown (third and fourth grade, possibly 9-10yrs) **Sampling procedure:** Noisy and quiet schools matched for age, grade, ethnic and racial distribution, families with assistance,	**Noise exposure**: aircraft noise levels **measured inside** the classroom for 1 hour in the morning and in the afternoon **Noise source**: aircraft noise **Noise metrics**: peak level, L33, LAeq 1h (dBA)	**Comparator:** Children from noisy classrooms compared to children from noise-abated classrooms and to children from quiet schools. Longitudinal analyses of children who moved from noisy to noise-abated classroom in contrast to children who remained in	**Confounding**: Number of children in the family, grade in school, months enrolled in school, race, cognitive aptitude test, performance under ambient conditions	**Outcome**: **California Test of Basic Skills – reading and mathematics tests** (California Assessment Program, 1976) **Auditory discrimination test** – ability to discriminate between pairs of words (Wepman, 1958) **Distractibility test – a** crossing out Es test under ambient and distracting conditions (Cohen et al, 1980)	**Findings:** Longitudinal testing: children from **noisy schools** were less distractible; more often failed solving the puzzles, solved puzzles for longer time; and had higher levels of noise perception than children in quiet schools. Cross-sectional: children from noisy classrooms more often failed the puzzle test in comparison to	**Comments: Findings:** No effect of noise on reading achievement or auditory discrimination between classrooms. No impact of noise on distraction between noisy, abated and quiet classrooms.

		parental educational and occupational level<br>**Exclusion criterion**: hearing impairment (audiometric screening)<br>**Longitudinal study;** follow-up period: 1 year (1977-1978)<br>**Cross-sectional study:** comparison between noisy, noise-abated and quiet classrooms		noisy rooms.		**Helplessness** – tested by speed of solving a solvable puzzle after trying to solve an unsolvable puzzle<br>**Perception of noise** – questionnaire for children	children from noise-abated and quiet rooms.<br>**Type of analyses:** Regression analyses<br>**Sample size relating to the effect size:** n=163 for longitudinal analysis, i.e. n=83 from noisy schools and n=80 from quiet schools<br>n=262 for cross-sectional analysis, i.e. n=97 from noisy, n=45 from noise-abated, and n=120 from quiet classrooms	
7.	Cohen et al, American Scientist, 1981, see Cohen et al above.							
8.	Matsui et al, Noise Health, 2004	**Population**: school children, high aircraft noise exposure at school, three groups of aircraft noise exposure	**Noise exposure**: aircraft noise levels at home and at school **modelled**<br>**Noise source**: aircraft noise	**Comparator**: children from homes with low noise levels (LAeq, 16h < 63 dB) compared to	**Confounding:** Age, sex, schools, household deprivation score (including income, home tenure, car ownership,	**Outcome**:<br>**Reading comprehension** Suffolk Reading Scale Level 2 (Hagley, 1987);<br>**Long term**	**Findings:**<br>**Aircraft** noise levels at home significantly predict poor performance at immediate recall	**Findings:**<br>No association between aircraft noise levels at home and delayed recognition,

		at home; total n=451, aged 8-9yrs, response rate: 83% in high noise schools and 81% in low noise schools<br>**Cross sectional** study	**Noise metrics**: LAeq, 16h (dB)<br>**Noise groups**: All children attending schools with high levels of aircraft noise (LAeq, 16h > 63 dB), but with different levels of aircraft noise at home (LAeq, 16h < 63dB vs. LAeq, 16h =63-66 dB, vs. LAeq, 16h > 63 dB)	children from schools with higher noise levels (LAeq, 16h =63-66 dB), and to the highest noise levels (LAeq, 16h > 63 dB)	employment, central heating, household crowding, social class), mother's educational level, long-standing illness, main language spoken at home	**Memory Recall and Recognition** – tested with Children's Memory scale (Cohen, 1997);<br>**Sustained Attention** tested with Tests of Everyday Attention for Children (TEA-Ch) (Manly et al, 1998)	and delayed recall tests.<br>**Type of analyses:** Multiple logistic regression analyses<br>**Sample size relating to the effect size:** n=163 with complete data	sustained attention and reading comprehension.
9.	Van Kempen et al, Enviro Health, 2010	**Population**: school children from 24 schools around Amsterdam airport, 620 children approached, total sample n=553, aged 9-11yrs<br>**Cross-sectional** study	**Noise exposure**: home and school addresses for each children linked to **modelled** noise levels<br>**Noise source**: aircraft and road traffic<br>**Noise metrics:** LAeq 7-23h (dBA) for aircraft noise; LAeq 7-23h (dBA) for road traffic noise	**Compared to:** lower levels of noise exposure	**Confounding**: Age, gender, main language spoken at home, long-standing illness, parental support for school work, school glazing, socio-economic status (crowding, home ownership, parental employment, mother's education), the other noise source	**Outcome**:<br>1) **Neurobehavioral evaluation system (NES) tests** (Letz, 1001): Simple Reaction Time Test, Switching Attention Test, Hand-Eye-Coordination Test, Symbol-Digit Substitution Test, Digit Memory Span Test.<br>2) **Other cognitive outcomes**: Reading comprehension (CRIE test, Staphorsius, 1994); Prospective	**Findings:** Positive and significant relation between **aircraft** noise exposure at school and the number of errors on Switching Attention test (SAT).<br>Positive and significant relation between **road traffic** noise at school and the number of errors on SAT test<br>**Type of analyses**: multilevel model analyses by MIXED procedure	**Comments:**<br>**Findings:** Significant correlation between aircraft and road traffic noise levels.<br>No relation between aircraft noise exposure at school and other NES tests; no relation between aircraft noise exposure at home and cognitive outcomes.<br>No relation between road traffic noise exposure at

						memory (writing initials where instructed); Episodic memory (Child Memory scale, Cohen, 1997); Working memory (Search and Memory test, Hygge et al, 2003); Sustained attention (Toulouse Pieron test, Toulouse et al, 1986)	**Sample size relating to the effect size:** n=433 children with complete data	home and cognitive outcomes.
10.	Van Kempen et al, Enviro Res, 2012	**Population**: school children from 24 schools around Amsterdam airport, 620 children approached, total sample n=553, aged 9-11yrs **Cross-sectional** study	**Noise exposure**: home and school addresses for each children linked to **modelled** noise levels **Noise source**: aircraft and road traffic **Noise metrics:** LAeq 7-23h (dBA) for aircraft noise; LAeq 7-23h (dBA) for road traffic noise	**Compared to:** lower levels of noise exposure	**Confounding**: Modelled concentrations of **NO2** (µg/m3) and particulate matter (**PM10**); Age, gender, main language spoken at home, long-standing illness, parental support for school work, school glazing, socio-economic status (crowding, home ownership, parental employment, mother's education)	**Outcomes: Neurobehavioral evaluation system (NES) tests** (Letz, 1001): Simple Reaction Time Test, Switching Attention Test, Hand-Eye-Coordination Test, Symbol-Digit Substitution Test, Digit Memory Span Test.	**Findings:** Positive and significant relation between **road traffic** noise at school and the number of errors on SAT test; Positive and significant relation between **aircraft** noise at school and the number of errors on SAT test; both significant after adjustment for NO2 concentration at school. **Type of analyses**:	**Comments: Findings:** Positive correlation between aircraft and road traffic noise levels at home and school; positive correlation between NO2 and PM10 at home and school. NO2 level at school significantly associated with span length measured with DMST. No relation

							multilevel model analyses by MIXED procedure **Sample size relating to the effect size:** n=485 children with complete data	between aircraft and road traffic noise exposure at home and cognitive outcomes. Combined exposure to NO2 and road traffic noise had effect on reaction times measured with SRTT and the number of errors on SAT.
**ROAD TRAFFIC NOISE EXPOSURE**								
**Cross-sectional Evidence**								
	See Van Kempen et al, Enviro Health, 2010 above							
	See Van Kempen et al, Enviro Res, 2012 above							
	See Stansfeld et al, Lancet, 2005 above							
11.	Sanz et al, Int Arch Enviro Health, 1993	**Population**: school children, two schools of similar socio-economic characteristics, total n=136, aged 6-11yrs.	**Noise exposure**: ambient noise levels **measured** outside and inside schools (classroom with closed windows) **Noise source**:	**Comparison**: Children from a high-noise school compared to children from a low-noise school	**Confounding:** None	**Outcomes:** **Attention** – Difference Perception Test (“face test”) (Tecnicos Expecialistes Asociados SA,	**Findings:** No difference between schools on the “face test”. Third-grade pupils from a high-noise school had poorer result	**Comments:** **Findings:** No adjustment for age, gender, etc reported in the paper.

		**Cross-sectional** study	road-traffic noise **Noise metrics**: LAeq (dBA) Noise groups: low-noise school (mean=54.4 dBA) vs. high-noise school (mean=57.9 dBA)			1973) and crossing out letters from a text (“text test”)	on “text test”. **Type of analyses:** Chi-square, t-test **Sample size relating to the effect size:** n=136	
12.	Cohen et al, J Experim Soc Psychol, 1973	**Population**: school children, total n=54, 2nd, 3rd, 4th and 5th grade (age not specified); **Sampling procedure**: 73 children approached, exclusion criteria: poor knowledge of English, not living at the building of interest, absence, disturbance on testing day, hearing deficit at audiometric test. **Cross sectional** study	**Noise exposure**: noise levels were **measured** outside the buildings, inside the buildings (hallways), and inside of the apartments (living room) **Noise source**: road traffic **Noise metrics**: not specified (dBA) **Noise groups:** children who lived in the noisy buildings ≥4 years, children who lived in noisy buildings ≤ 3 years	**Comparator**: Children who lived in noisy buildings ≥4 years were compared to children who lived in noisy buildings ≤ 3 years	**Confounding**: Audiometric testing, parent’s educational level, floor level, length of residence, school grade, number of children in family	**Outcome**: **Auditory discrimination** – measured by Auditory Discrimination test (Wepman, 1958); **Reading – including word vocabulary, reading comprehension and reading total** tested by the Metropolitan Achievement Reading Test (Durost et al, 1971); **Stroop Color-word test** – Uleman and Reeves, 1971	**Findings:** Children living in **noisy apartments** ≥4 years had positive correlation between auditory discrimination and reading test, positive correlation between floor level and auditory discrimination (adjustment for parent’s education); positive correlation between floor and reading test scores (adjustment for parent’s	**Comments:** **Methods:** Floor level as a surrogate of noise exposure. **Findings:** Duration of residence is related to impairment in auditory discrimination. Floor level accounts for the variance of auditory discrimination. Auditory discrimination and mother’s education account for the total variance in reading test score. **Conclusion:** The role of noise

							education). **Type of analyses:** Correlation, partial correlation, stepwise regression analysis **Sample size relating to the effect size:** n=54 (n=34 living ≥4 years, n=20 living ≤ 3 years in noisy buildings)	exposure in reading impairment is indirect (through auditory discrimination).
**RAILWAY NOISE EXPOSURE**								
**Cross-sectional Evidence**								
13.	Lercher et al, Enviro and Behav, 2003	**Population**: school children, total n=123, aged 9-10yrs. **Selection**: two neighborhoods selected from larger sample based on noise levels **Cross-sectional** study	**Noise exposure**: not specified whether noise levels were measured or modeled **Noise source**: rail and road traffic noise **Noise metrics**: Ldn **Noise groups**: noisy neighborhood (Ldn=62 dBA), and quiet neighborhood (Ldn=46.1 dBA)	**Comparison:** Children exposed to higher noise levels compared to children exposed to lower levels	**Confounding:** none	**Outcomes: Attention** – visual search task; **Intentional, explicit memory** – reading a story (Seyfried, 1998); **Incidental memory** – free recall; **Motivation** – solving insolvable and solvable puzzle (Evans et al, 2001)	**Findings:** No effect of noise on visual search performance (attention). Children from high noise group had poorer results on explicit and incidental memory tests. **Type of analyses:** Chi-square, t-test **Sample size relating to the effect size:** n=123	**Comments: Findings:** No adjustment for age, gender, parent's education, housing characteristics etc reported in the paper.

Table S5: Characteristics of included studies for environmental noise effects on executive function deficit (working memory)

	Reference	**Population**: general population in settings (hospitals, residences, public venues, educational facilities) + **response rate** and other **selection** /bias factors **Cross-sectional or longitudinal**	**Exposure**: exposure to high levels of environmental noise from various noise sources + noise metric involved + modelled or measured noise	**Comparator**: no noise exposure or lower levels of noise exposure	**Confounding**: adjusted for confounding	**Outcome**: assessment of outcome	**Findings:** expressed as effect per dB if possible. Type of analyses Sample size relating to the effect size	**Comments:** Anything else to note
**AIRCRAFT NOISE EXPOSURE**								
**CROSS-SECTIONAL EVIDENCE**								
1.	Haines, Stansfeld, Brentnall et al, Psychol Med, 2001	**Population**: school children, 20 schools, total n=451, aged 8-11yrs. Noisy and quiet schools matched for age, sex, other noise sources, noise protection at school, socio-economic status, main language; Response rate 82% **Cross-sectional study**	**Noise exposure**: aircraft noise levels **modelled** around schools **Noise source**: aircraft noise **Noise metrics**: LAeq, 16h (dBA) **Noise groups**: noisy schools (Leq, 16h >63 dBA), quiet schools (Leq, 16h <57 dBA)	**Comparator**: Children from high aircraft noise schools compared with children from lower aircraft noise level schools	**Confounding**: Household deprivation score – incorporating income, home tenure, car ownership, employment, central heating, social class and household crowding (Townsend, et al, 1989), age, main language spoken at home	**Outcome**: **Reading comprehension** – Suffolk Reading Scale (SRS2) Level 2 (Hagley, 2002); **Long-term memory** – including immediate recall, delayed recall, and recognition – adapted from Child Memory Scale (Cohen, 1997); **Backward serial digit recall** (Pickering and	**Findings:** Children in high noise schools had poor performance on the difficult items (20% of all items) of the reading scale. **Type of analyses:** Analysis of covariance (ANCOVA), Multilevel modeling analysis **Sample size relating to the effect size:** n=451 (n=236 from	**Comments:** **Findings:** No difference in reading, immediate recall, delayed recall, recognition memory, sustained attention and serial backward digit recall between children from high-level aircraft noise schools and children from low level noise schools.

						Gathercole, 2000); **Sustained Attention** – score task from Tests of Everyday Attention for Children (TEA-Ch) (Manly et al, 1998)	high-level noise schools, n=215 from low-level noise schools)	
2.	Haines, Stansfeld, Job et al, Psychol Med, 2001	**Population**: school children, 8 schools, total n=340, aged 8-11yrs. Noisy and quiet schools matched for age, sex, other noise sources, noise protection at school, socio-economic status, ethnic groups; Response rate 77% **Cross-sectional study**	**Noise exposure**: aircraft noise levels **modelled** around schools; acute noise levels **measured** indoors **Noise source**: aircraft noise **Noise metrics**: LAeq, 16h (dBA) outdoors; single event noise exposure level (SEL dBA); LAeq during time needed for task completion **Noise groups**: noisy schools (Leq, 16h >66 dBA), quiet schools (Leq, 16h <57 dBA)	**Comparator**: Children from high aircraft noise schools compared with children from lower aircraft noise level schools	**Confounding**: Household deprivation score – incorporating income, crowding, home ownership, unemployment, (Townsend, et al, 1989), age, main language spoken at home	**Outcome**: **Reading comprehension** – Suffolk Reading Scale (SRS2) Level 2 (Hagley, 2002); **Long-term memory** – including recognition task and recall task (Evans et al, 1995); **Working memory** – serial digit recall task; **Motivation – performance measure**, i.e. solving insolvable and solvable puzzle (Evans et al, 1995); **Child Attributional Style** – measured by Child Attribution Style Questionnaire (CASQ; Kaslow & Nolen-Hoeksema, 1991); **Classroom**	**Findings:** Children in high noise schools had poor performance on the reading scale than children from low noise schools (even after adjustment for deprivation, age, language). Children in high noise schools had poor long-term memory than children from low noise schools (only a subsample without bias). No effect of noise exposure on motivation, self-reported attributional scale and classroom motivation. **Type of analyses:** Analysis of	**Comments:** **Findings:** Acute aircraft noise exposure at the time of testing was not associated with reading comprehension or annoyance. **Conclusion:** The association between aircraft noise exposure and reading comprehension is independent from noise annoyance, acute noise levels, age, language, and deprivation.

						**motivation, i.e. learned helplessness** – estimated by teachers using Student Behaviour Checklist (SBC; Fincham et al. 1989)	covariance (ANCOVA) **Sample size relating to the effect size:** n=340 (n=169 from high-level noise schools, n=171 from low-level noise schools)	
3.	Stansfeld et al, Lancet, 2005	**Population**: school children, 89 schools around three airports, total n=2844, aged 9-13yrs. **Cross sectional** study	**Noise exposure**: aircraft noise levels at home and at school **measured** in an area from 7 am to 11 pm; road traffic noise at school **modelled** or **combination** of measurements with models **Noise source**: aircraft and road traffic **Noise metrics**: LAeq, 16h (dBA)	**Comparator**: lower levels of noise exposure	**Confounding**: age, gender, parental employment, home ownership, home crowding, mother's educational attainment, long-standing illness, main language spoken at home, parental support for school work, classroom glazing	**Outcome**: **Reading comprehension** Suffolk Reading Scale Level 2 (SRS2) (Hagley, 2002), CITO Readibility Index for elementary and Special Education (Staphorsius, 1994), Ecaluacion Comprension Lectora ECL-2 (De La Cruz, 1999); **Episodic memory** – Child Memory scale, (Cohen, 1997); **Sustained attention** – Toulouse Pieron test (Toulouse et al, 1986); **Working memory** – Search and Memory test (Smith and Miles, 1987; Hygge et al, 2003);	**Findings:** Increase of **aircraft** noise at school by 1 dBA correlated to decrease of the performance on the reading test by 0.008 marks; Increase of aircraft noise at school by 1 dBA correlated to decrease of the performance on the recognition test by 0.018 marks. Increase of **road traffic** noise by 1 dB was associated with an increase of information recall by 0.038 marks and an increase of conceptual recall	**Comments:** **Findings:** No association between aircraft and road traffic noise and cued conceptual recall, cued information recall, prospective memory, working memory, and sustained attention.

						**Prospective memory** (writing initials as instructed)	by 0.013 marks.<br>**Type of analyses:** Multilevel model analyses (for data clustering)<br>**Sample size relating to the effect size:** range from 1939 to 2014 with complete data	
4.	Stansfeld, Hygge, Clark et al, Noise Health, 2010	**Population**: school children, two studies re-analysed.<br>**Munich study**: n=326, aged 9-11yrs, around old and new airport<br>Inclusion criteria: 2 years of residence, speaking German fluently<br>**Longitudinal study;** baseline (wave 1): 6 months before the opening of new airport, follow-up 1 and 2 years (waves 2 and 3) after changeover of airports<br>**RANCH study:** n=857, aged 9-10yrs, around	**Noise exposure**: aircraft noise levels **measured** around schools in Munich study; **modeled** in RANCH study<br>**Noise source**: aircraft noise<br>**Noise metrics**: Munich study: Leq, 24h (dBA);<br>RANCH study: Leq, nighttime at home (dBA) (11 p.m. to 7 a.m.)	**Comparison**: Munich study: children from noisy schools around the old and the new airport compared to children from quiet schools around the old and the new airport.<br>RANCH study: higher levels of aircraft noise exposure compared to lower levels of noise	**Confounding**: Munich study: self-reported sleep quality; RANCH study: daytime aircraft noise at school, road-traffic noise, sleep problems, age, sex, parental employment, crowding, homeownership, mother's education, child's illness, main language at home, parental support for home work, classroom glazing	**Outcomes:**<br>**Munich study: Reading test** in German (Biglmaier, 1969) in noisy conditions (80 dBA)<br>**Long-term memory** – recall of a text read in noisy conditions the previous day.<br>**RANCH study: Reading comprehension** Suffolk Reading Scale Level 2 (SRS2) (Hagley, 2002),<br>**Episodic memory** – Child Memory Scale, (Cohen, 1997); **Sustained attention** – Toulouse Pieron test (Toulouse et al, 1986);	**Findings:**<br>**Munich study:** No association between **nighttime aircraft** noise and cognitive impairment (mediated by sleep quality).<br>**RANCH study:** Increase of **nighttime aircraft** noise at home by 1 dBA correlated to decrease of the reading test performance by 0.009 marks, and to decrease of recognition memory by 0.031 marks.<br>**Type of analyses:** Multilevel modeling<br>**Sample size**	**Comments:**<br>**Findings:**<br>RANCH study: Daytime and nighttime noise exposure highly correlated.<br>Nighttime aircraft exposure was no longer associated with impaired reading comprehension and recognition memory after adjustment for daytime aircraft noise at school.

		the airport **Cross-sectional** study				**Working memory** – Search and Memory test (Smith and Miles, 1987; Hygge et al, 2003)	**relating to the effect size:** n=326 (Munich study), n=842 for RANCH study	
5.	Clark et al, Am J Epidemol, 2012	**Population**: school children, total n=960, aged 9-10yrs. **Cross sectional** study	**Noise exposure**: aircraft noise levels at home and at school **measured** in an area from 7 am to 11 pm; road traffic noise at school **combined** from measurements and models **Noise source**: aircraft and road traffic **Noise metrics**: LAeq, 16h (dBA)	**Comparator**: lower levels of noise exposure	**Confounding**: Modelled concentrations of **NO2** (μg/m3); Socio-economic status (employment, housing tenure, home crowding), maternal education, ethnicity, main language spoken at home; age, gender, long-standing illness, parental support for schoolwork, classroom glazing; other noise exposure source	**Outcome**: **Reading comprehension** Suffolk Reading Scale Level 2 (SRS2) (Hagley, 2002), CITO Readibility Index for elementary and Special Education (Staphorsius, 1994), Ecaluacion Comprension Lectora ECL-2 (De La Cruz, 1999); **Episodic memory** tested with Child Memory scale, (Cohen, 1997) **Working memory** (Search and Memory test, Hygge et al, 2003).	**Findings:** Increase of **aircraft** noise at school by 1 dBA correlated to decrease of the performance on the following tests: reading comprehension (by 0.01 marks), recognition memory (by 0.045 marks), information recall (by 0.043 marks) and conceptual recall (by 0.015 marks). **Type of analyses:** Multilevel linear and logistic regression models **Sample size relating to the effect size:** n=719 with air pollution data and n=241 without air pollution data	**Comments:** **Findings:** Moderate correlation between NO2 levels and aircraft noise and road traffic noise. No association between road traffic noise and reading comprehension
6.	Matheson et al,	**Population**:	**Noise exposure**:	**Comparator**:	**Confounding**:	**Outcome**:	**Findings:**	**Comments:**

	Noise Health, 2010	school children, 89 schools around three airports, total n=2844, aged 9-13yrs.<br>**Sampling**: 3207 children approached; 2844 pupils participated; response rate: 89% of children, 80% of parents<br>**Cross sectional** study	aircraft noise levels at home and at school **measured** or **modeled** in an area; road traffic noise at school **combined** from measurements and models; acute noise measurements<br>**Noise source**: aircraft and road traffic<br>**Noise metrics**: LAeq, 16h (dBA)	lower levels of noise exposure	age, gender, country, socio-economic status (employment, home ownership, home crowding), mother's educational attainment, long-standing illness, main language spoken at home, parental support for school work, other noise source; dyslexia, acute noise during testing	**Episodic memory** (including information recall, conceptual recall, recognition memory) tested with Children's Memory scale (Cohen, 1997);<br>**Prospective memory** test (Shield and Dockrell, 2002)<br>**Working memory** (Search and Memory test, Hygge et al 2003).	Increase of **road traffic** noise by 1 dB was associated with an increase of information recall by 0.038 marks and an increase of conceptual recall by 0.013 marks.<br>Increase of **aircraft** noise by 1 dB was associated with a decrease of recognition memory by 0.018 marks but was not related to prospective memory.<br>**Type of analyses:** Multilevel linear regression analyses<br>**Sample size relating to the effect size:** n=2844 with complete data	**Findings:** Aircraft noise was not significantly related to either information recall or conceptual recall. Road traffic noise was not significantly related to either recognition memory or prospective memory.
7.	Van Kempen et al, Enviro Health, 2010	**Population**: school children from 24 schools around Amsterdam airport, 620 children approached,	**Noise exposure**: home and school addresses for each children linked to **modelled** noise levels<br>**Noise source**: aircraft and road	**Compared to:** lower levels of noise exposure	**Confounding**: Age, gender, main language spoken at home, long-standing illness, parental support for school work, school glazing,	**Outcome**:<br>1) **Neurobehavioral evaluation system (NES) tests** (Letz, 1001): Simple Reaction Time Test, Switching	**Findings:** Positive and significant relation between **aircraft** noise exposure at school and the number of errors	**Comments:**<br>**Findings:** Significant correlation between aircraft and road traffic noise levels.<br>No relation

		total sample n=553, aged 9-11yrs<br>**Cross-sectional** study	traffic<br>**Noise metrics:** LAeq 7-23h (dBA) for aircraft noise; LAeq 7-23h (dBA) for road traffic noise		socio-economic status (crowding, home ownership, parental employment, mother's education), the other noise source	Attention Test, Hand-Eye-Coordination Test, Symbol-Digit Substitution Test, Digit Memory Span Test.<br>2) **Other cognitive outcomes**: Reading comprehension (CRIE test, Staphorsius, 1994); Prospective memory (writing initials where instructed); Episodic memory (Child Memory scale, Cohen, 1997); Working memory (Search and Memory test, Hygge et al, 2003); Sustained attention (Toulouse Pieron test, Toulouse et al, 1986)	on Switching Attention test (SAT).<br>Positive and significant relation between **road traffic** noise at school and the number of errors on SAT test<br>**Type of analyses**: multilevel model analyses by MIXED procedure<br>**Sample size relating to the effect size:** n=433 children with complete data	between aircraft noise exposure at school and other NES tests; no relation between aircraft noise exposure at home and cognitive outcomes.<br>No relation between road traffic noise exposure at home and cognitive outcomes.
8.	Van Kempen et al, Enviro Res, 2012	**Population**: school children from 24 schools around Amsterdam airport, 620 children approached, total sample n=553, aged 9-	**Noise exposure**: home and school addresses for each children linked to **modelled** noise levels<br>**Noise source**: aircraft and road traffic<br>**Noise metrics:**	**Compared to:** lower levels of noise exposure	**Confounding**: Modelled concentrations of **NO2** (μg/m3) and particulate matter (**PM10**);<br>Age, gender, main language spoken at home, long-standing illness,	**Outcomes:**<br>**Neurobehavioral evaluation system (NES) tests** (Letz, 1001): Simple Reaction Time Test, Switching Attention Test, Hand-Eye-Coordination Test,	**Findings:**<br>Positive and significant relation between **road traffic** noise at school and the number of errors on SAT test;<br>Positive and significant	**Comments:**<br>**Findings:**<br>Positive correlation between aircraft and road traffic noise levels at home and school; positive correlation

		11yrs **Cross-sectional** study	LAeq 7-23h (dBA) for aircraft noise; LAeq 7-23h (dBA) for road traffic noise		parental support for school work, school glazing, socio-economic status (crowding, home ownership, parental employment, mother's education)	Symbol-Digit Substitution Test, Digit Memory Span Test.	relation between **aircraft** noise at school and the number of errors on SAT test; both significant after adjustment for NO2 concentration at school. **Type of analyses**: multilevel model analyses by MIXED procedure **Sample size relating to the effect size:** n=485 children with complete data	between NO2 and PM10 at home and school. NO2 level at school significantly associated with span length measured with DMST. No relation between aircraft and road traffic noise exposure at home and cognitive outcomes. Combined exposure to NO2 and road traffic noise had effect on reaction times measured with SRTT and the number of errors on SAT.
9.	Klatte et al, Report, NORAH study, 2014	**Population**: 85 second-grade classes from 29 primary schools around Frankfurt/Main airport; children: total n=1243, age 8yrs, 4months **Cross-sectional** study	**Noise exposure:** Noise contours at school and at home around airport **Noise source:** aircraft noise **Noise metrics:** Leq around schools (time 8-14h); average 49.5 dB, Median 50.6 dB; range 39 to 59 dB;	**Comparison:** Change estimate per 10 dBA increase	**Confounding:** Migrant background. Children´s exposure to road traffic and railway noise. Also used as confounders: auditory thinking, phonological awareness, episodic memory. Class	**Outcomes:** **Reading test**: standardized comprehension test for primary school children (ELFE 1-6, Lenhard & Schneider, 2006). **Language functions** – speech perception, auditory thinking,	**Findings:** A 10 dB increase in aircraft noise associated with a decrement of one-tenth of an SD on the **reading** test, corresponding to a one month reading delay in this test. Similar results for	**Comments:** **Methods:** Impossible to separate home and schools exposure (high correlation). **Findings:** Significant confounding of migrant background – aircraft noise

			Leq at home (6-18h)<br>**Noise groups:**<br>3 groups at school: low exposure < 47 dB; middle exposure 47 to < 55 dB; high exposure ≥ 55 dB		characteristics (size, proportion of migrants, socioeconomic status, parental engagement), classroom reverberation and insulation.	phonological short-term memory, phonological awareness, episodic memory test.<br>**Other outcomes**: Health-related quality of life, well-being at home and school, noise annoyance at home and school.	subscales: word and text comprehension. Similar results found for aircraft noise at home.<br>**Type of analyses:** Multilevel regression<br>**Sample size relating to the effect size:** n=1090	(both home and school) strongly affects **reading** comprehension of children without migration background (1.5-2.5 months reading delay).<br>No effect of aircraft noise on tested **language** functions.<br>Aircraft noise at school and at home significantly associated with **annoyance**.<br>Aircraft noise was associated with less positive judgments of health-related **quality of life**, well-being at school, and sleep quality.
